# DAF-16 and TCER-1 Facilitate Adaptation to Germline Loss by Restoring Lipid Homeostasis and Repressing Reproductive Physiology in *C*. *elegans*

**DOI:** 10.1371/journal.pgen.1005788

**Published:** 2016-02-10

**Authors:** Francis Raj Gandhi Amrit, Elizabeth Marie Steenkiste, Ramesh Ratnappan, Shaw-Wen Chen, T. Brooke McClendon, Dennis Kostka, Judith Yanowitz, Carissa Perez Olsen, Arjumand Ghazi

**Affiliations:** 1 Department of Pediatrics, Rangos Research Center, University of Pittsburgh School of Medicine, Pittsburgh, Pennsylvania, United States of America; 2 Division of Basic Sciences Fred Hutchison Cancer Research Center, Seattle, Washington, United States of America; 3 Department of Obstetrics, Gynecology and Reproductive Sciences, Magee Women’s Research Institute, University of Pittsburgh School of Medicine, Pittsburgh, Pennsylvania, United States of America; 4 Departments of Developmental Biology and Computational and Systems Biology, Rangos Research Center, University of Pittsburgh School of Medicine, Pittsburgh, Pennsylvania, United States of America; University of California San Francisco, UNITED STATES

## Abstract

Elimination of the proliferating germline extends lifespan in *C*. *elegans*. This phenomenon provides a unique platform to understand how complex metazoans retain metabolic homeostasis when challenged with major physiological perturbations. Here, we demonstrate that two conserved transcription regulators essential for the longevity of germline-less adults, DAF-16/FOXO3A and TCER-1/TCERG1, concurrently enhance the expression of multiple genes involved in lipid synthesis and breakdown, and that both gene classes promote longevity. Lipidomic analyses revealed that key lipogenic processes, including *de novo* fatty acid synthesis, triglyceride production, desaturation and elongation, are augmented upon germline removal. Our data suggest that lipid anabolic and catabolic pathways are coordinately augmented in response to germline loss, and this metabolic shift helps preserve lipid homeostasis. DAF-16 and TCER-1 also perform essential inhibitory functions in germline-ablated animals. TCER-1 inhibits the somatic gene-expression program that facilitates reproduction and represses anti-longevity genes, whereas DAF-16 impedes ribosome biogenesis. Additionally, we discovered that TCER-1 is critical for optimal fertility in normal adults, suggesting that the protein acts as a switch supporting reproductive fitness or longevity depending on the presence or absence of the germline. Collectively, our data offer insights into how organisms adapt to changes in reproductive status, by utilizing the activating and repressive functions of transcription factors and coordinating fat production and degradation.

## Author Summary

The balance between production and breakdown of fats is critical for health, especially during reproduction-related changes such as onset of puberty or menopause. However, little is known about how animals retain a balanced metabolism when undergoing major life events. Here, we have used a *C*. *elegans* mutant that successfully adapts to loss of reproductive cells to address this question. Our data suggest that the conserved proteins DAF-16/FOXO3A and TCER-1/TCERG1 mediate a coordinated increase in fat synthesis and degradation when the reproductive cells are lost. This coupling likely helps the animal to manage the lipids that would have been deposited in eggs as yolk, thus preventing metabolic disarray. These proteins also inhibit processes that would have normally supported reproduction. Together the activities of these transcription regulators allow the mutant to convert a debilitating loss of fertility into improved health and longevity. We also report that TCER-1 promotes reproductive health in normal adults, whereas when procreation is impeded, it switches roles to repress fertility and enhance lipid equilibrium. These observations offer insights into how complex organisms coordinate their metabolism to suit their reproductive needs.

## Introduction

Organisms are constantly adapting to fluctuations in their internal and external milieus by employing transcriptional, translational and endocrine mechanisms that sense varying stimuli and activate or repress different cellular processes in response. But, how metazoans maintain metabolic homeostasis when faced with multidimensional challenges such as increasing age, altered reproductive status or competing physiological demands is poorly understood. Failure to execute metabolic adaptability under such conditions has come to be seen as the underlying cause of a host of human pathologies including age-related diseases such as diabetes and metabolic syndrome [1, 2].

The nematode *Caenorhabditis elegans* has proven to be a versatile platform for investigating aging and its modulation by various factors ranging from insulin IGF1 signaling (IIS) to dietary intake and reproduction. In *C*. *elegans*, germline loss increases lifespan and improves stress resistance [3, 4]. Similar observations have been made in other animals, including flies [5], mice [6], rats [7], grasshoppers [8] and salmon [9], and in some human population studies as well [10], suggesting that the reproductive control of aging may be conserved. But, this longevity is not just a byproduct of sterility because lifespan is extended only upon the removal of a specific population of totipotent germline-stem cells (GSCs) that are the precursors of the entire adult germline [5, 11]. GSCs must produce signals that coordinate reproductive status with rate of aging; upon their removal, the animal not only copes with the challenge of fertility loss, it also successfully reestablishes metabolic homeostasis and converts the drawback into a favorable lifespan increment. Mechanisms underlying this remarkable adaptability are poorly understood.

Transcriptional changes facilitated by modular gene-regulatory networks (GRNs) constitute a major mechanism by which animals respond to changing environmental conditions. In *C*. *elegans*, removal of GSCs in the gonad activates a network of conserved transcription factors in the intestine- a tissue that serves as the main adipose depot in worms and subsumes roles of the liver and pancreas. This network includes DAF-16/FOXO3A [12], PHA-4/FOXA [13], HLH-30/TFEB [14], SKN-1/NRF2 [15, 16], HSF-1/HSF1 [17] and nuclear hormone receptors (NHR), DAF-12/VDR [4], NHR-80/HNF4 [18] and NHR-49/ PPARα [19]. Many of these proteins, including DAF-16, are shared longevity determinants that alter lifespan in response to other interventions such as reduced IIS or dietary restriction (DR) [14, 20–22]. Previously, we identified TCER-1, the worm homolog of a human transcription elongation and splicing factor, TCERG1 [23], and showed that it specifically increases lifespan following germline loss, likely by facilitating a distinct pattern of DAF-16-dependent gene expression [24]. While DAF-16 targets have been identified in multiple contexts [25], little is known about downstream effectors of TCER-1. Additionally, studies so far have largely focused on genes upregulated following germline depletion, although, it is conceivable that adaptation to GSC loss also necessitates the downregulation of some genes. DAF-16, like most transcription factors, both activates and represses transcription, but its negative targets are not well characterized. Transcription-repression functions of TCERG1 have been documented [26] but genes repressed by TCER-1 are unknown. Thus, the elements of gene-expression networks orchestrated by DAF-16 and TCER-1 in GSC-less animals, the extent of their overlap with one another, and the molecular pathways governed by them are as yet unidentified.

Germline removal is accompanied by the enhancement of cellular processes that govern macromolecular homeostasis including proteasomal activity and autophagic flux [13, 14, 16]. Several lines of evidence have also indicated a crucial role for lipid metabolism in this longevity paradigm [27–30]. The lifespan extension of GSC-less animals is dependent on multiple components of a steroid signaling pathway that culminates in the production of bile acid-like steroids called dafachronic acids (DA) that control the activity of the NHR, DAF-12 [31]. NHR-80 mediates the increased expression of fatty-acid desaturases [18], and in a recent study, we demonstrated the importance of NHR-49/ PPARα in elevating fatty-acid β-oxidation and desaturation to promote longevity of germline-depleted worms [19]. Germline-less worms display elevated lipid levels [32], but paradoxically, a lipase, LIPL-4, has been found to be essential for their long lifespan [33], and the molecular basis of their dramatic fat accrual remains unknown. LIPL-4 promotes autophagy in germline-less mutants [13] and lipid signals appear to mediate SKN-1 activation [15], but overall, little is understood of the broader significance of lipid-metabolic pathways in the lifespan increment associated with germline depletion.

In this study, we set out to explore the GRNs governed by DAF-16 and TCER-1 following germline loss by identifying genes whose expression is modulated by the two factors. RNA-Sequencing (RNA-Seq)-based transcriptome mapping revealed that these proteins shared a significant fraction of their downstream targets. In studying these targets, we discovered that DAF-16 and TCER-1 augmented expression of genes involved in both lipid anabolism and catabolism. These gene-expression changes are biochemically and functionally relevant because our lipidomic analyses demonstrated that lipogenic processes are enhanced upon germline loss. Previous reports have shown increased lipase activity in these animals, and we found both classes of genes are important for germline-less longevity. Our observations suggest that germline loss may trigger the simultaneous enhancement of antagonistic lipid-metabolic pathways and this may enable adaptation to loss of reproductive capacity. We also found that DAF-16 and TCER-1 performed important repressive functions. Upon germline depletion, TCER-1 blocked the expression of somatic genes that facilitate reproduction as well as anti-longevity genes. DAF-16 suppressed translation by inhibiting the expression of genes encoding ribosomal subunits. In addition, we discovered an unexpected requirement for *tcer-1* in ensuring the reproductive fitness of normal, fertile adults. Overall, these experiments provide mechanistic insight into how an organism adapts to change in reproductive status and maintains metabolic equilibrium by modulating anabolic and catabolic processes.

## Results

### Mapping the transcriptomes governed by TCER-1 and DAF-16 upon germline loss

The lifespan extension resulting from germline elimination in *C*. *elegans* is faithfully simulated by mutations that cause GSC loss and sterility. One such temperature-sensitive mutant, *glp-1(e2141ts)*, has been used widely as a model for this longevity [11, 24]. To map the transcriptomes governed by DAF-16 and TCER-1 in germline-ablated, long-lived animals, we performed RNA-Seq on three strains (i) *glp-1(e2141ts)* (ii) *daf-16(mu86);glp-1(e2141ts)* and (iii) *tcer-1(tm1452);glp-1(e2141ts)* (henceforth referred to as *glp-1*, *daf-16;glp-1* and *tcer-1;glp-1*, respectively). These comparisons allowed us not only to document the transcriptional changes mediated by DAF-16 and TCER-1 upon GSC ablation, but also to assess the overlap between their targets. Since many molecular landmarks associated with germline-less longevity are observed once the animal becomes an adult [12, 24], and since DAF-16 and TCER-1 both act during adulthood to promote *glp-1* longevity [11], we isolated RNA from day 2 adults grown under identical conditions. Sequencing data was analyzed using the publicly available Galaxy pipeline running Tuxedo Suite tools [34] (S1A Table; see Methods for details).

**Fig 1:**
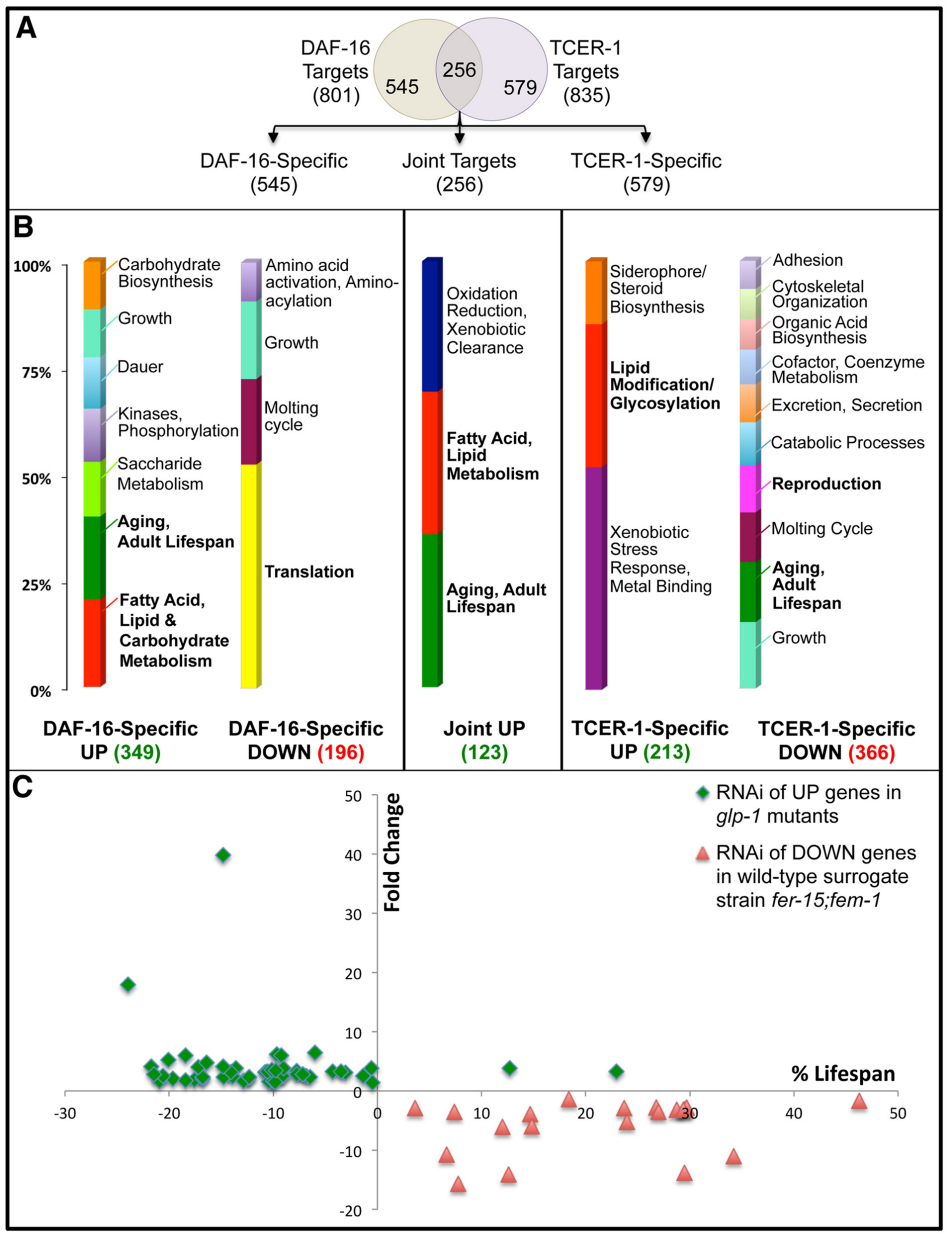
Identification of genes up regulated and downregulated by DAF-16 and TCER-1 following germline loss, and effects of their knockdown on lifespans of *glp-1* mutants and wild-type worms, respectively. **A.** Comparison of the transcriptomes of *glp-1*, *daf-16*;*glp-1* and *tcer-1*;*glp-1* day 2 adult mutants by RNA-Seq identified 685 genes whose expression is increased in *glp-1* mutants (UP genes) by DAF-16 and/or TCER-1 (349 DAF-16-Specific UP targets, 213 TCER-1-Specific UP targets and 123 Joint UP targets) and 635 genes whose expression was repressed (DOWN genes: 196 DAF-16-Specific, 366 TCER-1-Specific and 73 Joint). **B:** DAVID analysis of DAF-16 and TCER-1 targets. The three UP groups (green) share an enrichment of lipid-metabolic genes (labeled in bold). Aging and lifespan regulation is another notable category enriched in DAF-16-Specific and Joint UP groups. The major GO category enriched in DAF-16-Specific DOWN group was translation (bold), whereas, growth, reproduction and aging were highly enriched in the TCER-1-Specific DOWN class. **C:** RNAi knockdown of UP and DOWN genes shortens *glp-1* longevity and increases the lifespan of a wild-type surrogate strain *fer-15;fem-1*, respectively. The scatter-plot represents the combined results from knockdown of UP and DOWN genes. UP genes were inactivated by initiating RNAi from day 1 of adulthood in *glp-1* mutants (green diamonds). The red triangles represent RNAi inactivation of TCER-1-Specific DOWN genes, from day 1 of adulthood, in the strain *fer-15;fem-1* that has been used extensively as a surrogate for wild-type worms [16, 17]. The X-Axis depicts the fold-change in gene expression detected by RNA-Seq for each of the genes. The Y-Axis represents the percent effect on lifespan as compared to the strains grown on empty vector control. Data shown here is from experiments of Trial #1 in S3 and S8 Tables, and do not include joint DAF-16/TCER-1 targets (as they have two different fold-change values). See S3 and S8 Tables for detailed lifespan data.

We found that DAF-16 and TCER-1 mediated the transcriptional upregulation and downregulation of overlapping groups of target genes, henceforth designated UP and DOWN genes, respectively. The global comparison of *glp-1* and *daf-16;glp-1* identified 801 genes that were differentially expressed (‘DAF-16 Targets’) (Fig 1A and 1B; S1B, S1C and S1F–S1H Table) and comparison of *glp-1* and *tcer-1;glp-1* revealed 835 such genes (‘TCER-1 Targets’) (Fig 1A and 1B; S1D–S1H Table). 256 genes were shared between these two sets, constituting a newly identified group of genes regulated by both TCER-1 and DAF-16 (‘Joint Targets’) (Fig 1A; S1F–S1H Table). Of these ‘Joint Targets’, 123 genes were upregulated in both mutants (‘Joint UP’, S1F Table), 73 were downregulated (‘Joint DOWN’, S1G Table) and 60 showed opposite regulation (S1H Table). The overlap between DAF-16 and TCER-1 targets was 5.2 times greater than that expected by random chance and comprised significant portions of the total transcriptomes dictated by each regulator (DAF-16, 31.9% and TCER-1, 30.7%; p<2.041e-88). These findings are consistent with our earlier data that suggested that DAF-16 and TCER-1 function in the same genetic pathway to mediate the reproductive control of aging [24].

### Previously-identified DAF-16 ‘Consensus’ target genes are over-represented in the transcriptome of germline-less adults

There has been limited agreement between DAF-16 targets identified previously through other genomic approaches [25]. Through a meta-analysis of DAF-16-related genomic studies, Tepper *et al*., have arrived at a ‘Consensus’ list of DAF-16 positive and negative targets (corresponding to our UP and DOWN classes, respectively) [35]. We compared the DAF-16 targets obtained from our RNA-Seq analysis with these lists and found 162/349 DAF-16-Specific UP genes (46.4%, p<9.4e-80) and 70/123 Joint UP genes (56.9%, p<1.46e-42) to be shared with the DAF-16 ‘Consensus’ positive targets (S2 Table). Of the genes repressed by DAF-16, 110/196 DAF-16-Specific DOWN genes (56.1%; p<1.9e-63) and 41/73 Joint DOWN genes (56.1%, p<2.1e-24) were represented in the DAF-16 ‘Consensus’ negative target list (see S2 Table for details and additional comparisons). This degree of overlap is striking in the light of the dissonance observed between various DAF-16-target compilations [25]. In a recent analysis of individual DAF-16 isoforms, Chen *et al*., identified 399 targets of *daf-16a*, the transcript with the strongest impact on germline-less longevity [36]. 58 of DAF-16-Specific and 62 of the Joint UP targets were shared with this list (15%; p< 1.797e-59) (S2 Table). McCormick *et al*., reported the identification of 230 ‘DAF-16-regulated’ genes in a *glp-1* background [37]. 85 of these genes were represented among our DAF-16 targets (37%; p< 7.840e-55). In contrast, only 10 were included in the TCER-1 list (4%; p = 0.2) (S2 Table). Together, these comparisons reinforced our confidence in the newly-identified DAF-16 targets as well as the TCER-1 downstream genes. It is noteworthy that a majority of the DAF-16 targets we identified were novel, particularly in the *glp-1* background, underscoring the sensitivity of our approach.

### DAF-16 and TCER-1 UP targets are transcriptionally upregulated following germline loss and collectively promote *glp-1* longevity

Based on previous observations, we reasoned that the expression of UP genes is likely to be elevated upon germline removal. Indeed, when we compared the mRNA levels between wild type and *glp-1* adults using quantitative PCRs (Q-PCRs), 26/30 UP genes tested showed elevated expression in *glp-1* mutants (eight did not achieve statistical significance) and 22 of these depended on completely or partially DAF-16 and/or TCER-1 for their upregulation (Q-PCRs in this study). Following this substantiation of the RNA-Seq data, we asked if the increased expression of the UP genes was essential for *glp-1* mutants’ longevity. Using feeding RNAi, the levels of DAF-16-Specific, TCER-1-Specific and Joint UP targets were reduced in *glp-1* mutants. To obviate developmental phenotypes, RNAi was initiated on the first day of adulthood. Based on the fold change in expression, we tested the top 80 candidates and found that RNAi of 64 genes caused a statistically significant suppression of *glp-1* mutants’ longevity and 54 of these clones suppressed lifespan in at least two independent trials (Fig 1C and S3 Table). This group included genes encoding multiple transcription factors with roles in lifespan regulation (eg., *nhr-49*, *hlh-30*, *skn-1*) [14, 15, 19], previously-identified DAF-16 targets (eg., *mtl-1*) [38] as well as novel genes that implicated new cellular processes in influencing lifespan (see Discussion). Overall, these experiments demonstrated that our RNA-Seq analysis had identified DAF-16 and TCER-1 targets whose expression was elevated following germline removal and that were important for the consequent longevity.

### DAF-16 and TCER-1 activate specific lipid-metabolic pathways upon germline loss

**Fig 2:**
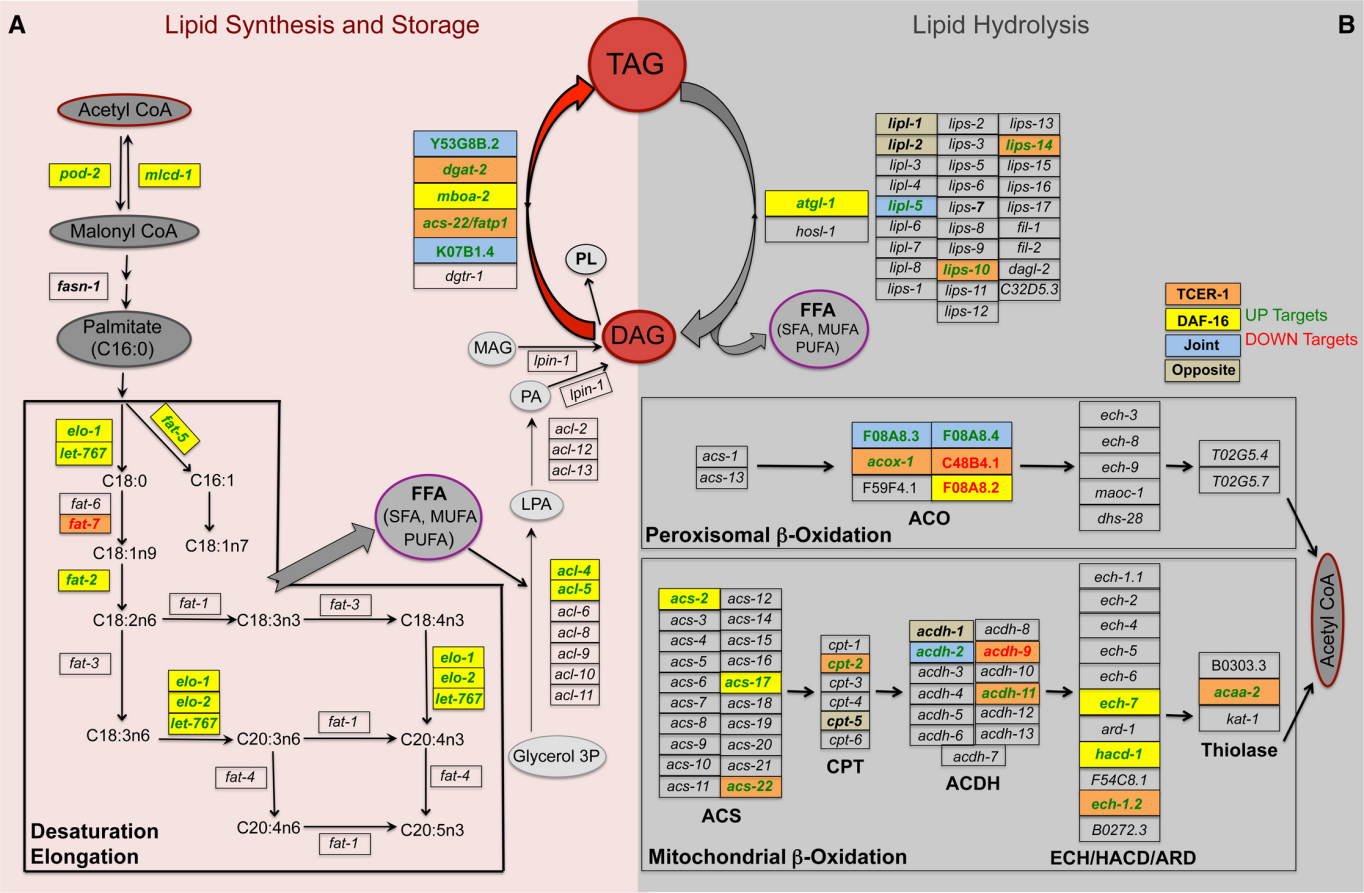
Key genes involved in lipid anabolism and catabolism are up regulated by DAF-16 and TCER-1 following germline loss. **A**. Lipid Synthesis: Overview of reactions involved in fatty-acid synthesis initiation, desaturation and elongation, and the steps leading up to triglyceride (TAG) formation. Acetyl CoA molecules, through the combined activities of the enzymes encoded by *pod-2*, *mlcd-1* and *fasn-1* are converted to the saturated fatty-acid (SFA) Palmitate (C16:0). Palmitate can be converted to longer mono- and poly-unsaturated fatty acids (MUFAs and PUFAs, respectively) by elongase and desaturase enzymes (encoded by *‘elo’* and *‘fat’* genes, respectively). These free fatty acids (FFAs) are joined with glycerol 3-phosphate (Glycerol 3P) to form lysophosphatidic acid (LPA) and phosphatidic acid (PA). PA and monoglycerides (MAG) serve as substrates for formation of diglycerides (DAG). DAGs are converted to the neutral, storage lipid TAGs by action of diacylglycerol acyl transferase (DGAT) enzymes. DAGs are also used in phospholipid (PL) production. **B.** Lipid Breakdown: Lipid degradation commences with the hydrolysis of TAGs into DAGs through the activity of ATGL-1, and other lipases and lipase-like enzymes (encoded by the ‘*lipl’* and ‘*lips’* genes). The FFAs released as a result are broken down to Acetyl CoA through peroxisomal- and mitochondrial- β-oxidation. Genes predicted to encode enzymes involved at different steps of these pathways are shown. ACO: acetyl CoA oxidase; ACS: acetyl CoA synthase; ACDH: acetyl CoA dehydrogenase; ECH: enoyl CoA hydratase; HACD: hydroxy acyl CoA dehydrogenase. Genes that were identified in the RNA-Seq analysis as DAF-16 and/or TCER-1 targets are highlighted in colored boxes as follows: DAF-16-Specific (yellow), TCER-1-Specific (orange) and Joint (blue). Genes up regulated by these proteins (UP) are written in green, whereas, DOWN targets are represented in red. Genes that were predicted to undergo opposite regulation by DAF-16 and TCER-1 are shown in olive boxes. Notably, *pod-2*, *mlcd-1* and 4/6 genes encoding DGAT enzymes were identified as UP targets. *K07B1*.*4* was not picked up by RNA-Seq but is highlighted because it was previously identified as a common target of DAF-16 and TCER-1 [24].

We performed DAVID analysis [39, 40] on the RNA-Seq data (S4 Table) and used REVIGO [41] to visualize the enriched GO term classes and their semantic similarities in two-dimensional space (S5 Table). DAF-16-Specific, TCER-1-Specific and Joint UP classes revealed a shared enrichment of fatty acid and lipid metabolism genes (Fig 1B; S4A, S4B, S4E, S4F, S4I and S5A, S5C and S5E Tables). A detailed examination of these gene lists revealed that (a) specific lipid-metabolic pathways were enriched in this data set and (b) these pathways included both lipid anabolic {initiation of fatty-acid synthesis, desaturation and elongation, and conversion of diglycerides (DAG) to triglycerides (TAG)} and catabolic (TAG hydrolysis and fatty-acid β-oxidation) processes (Fig 2). For instance, fifteen of the thirty genes predicted to collectively mediate fatty-acid synthesis and TAG production were represented in our RNA-Seq dataset (50%; p< 8.892e-06), fourteen of which were UP genes (Fig 2A). Similarly, of the 98 genes together predicted to be involved in TAG hydrolysis and fatty-acid β-oxidation, 24 were identified by RNA-Seq (25%, p<1.8e-4) and 17 of these were UP genes (Fig 2B). The over-representation of genes involved in fat buildup was particularly noteworthy and has not been reported before. It prompted us to investigate the role of DAF-16 and TCER-1 in modulating lipid-metabolic pathways.

### *de novo* fatty-acid synthesis is elevated in germline-less adults by DAF-16

**Fig 3:**
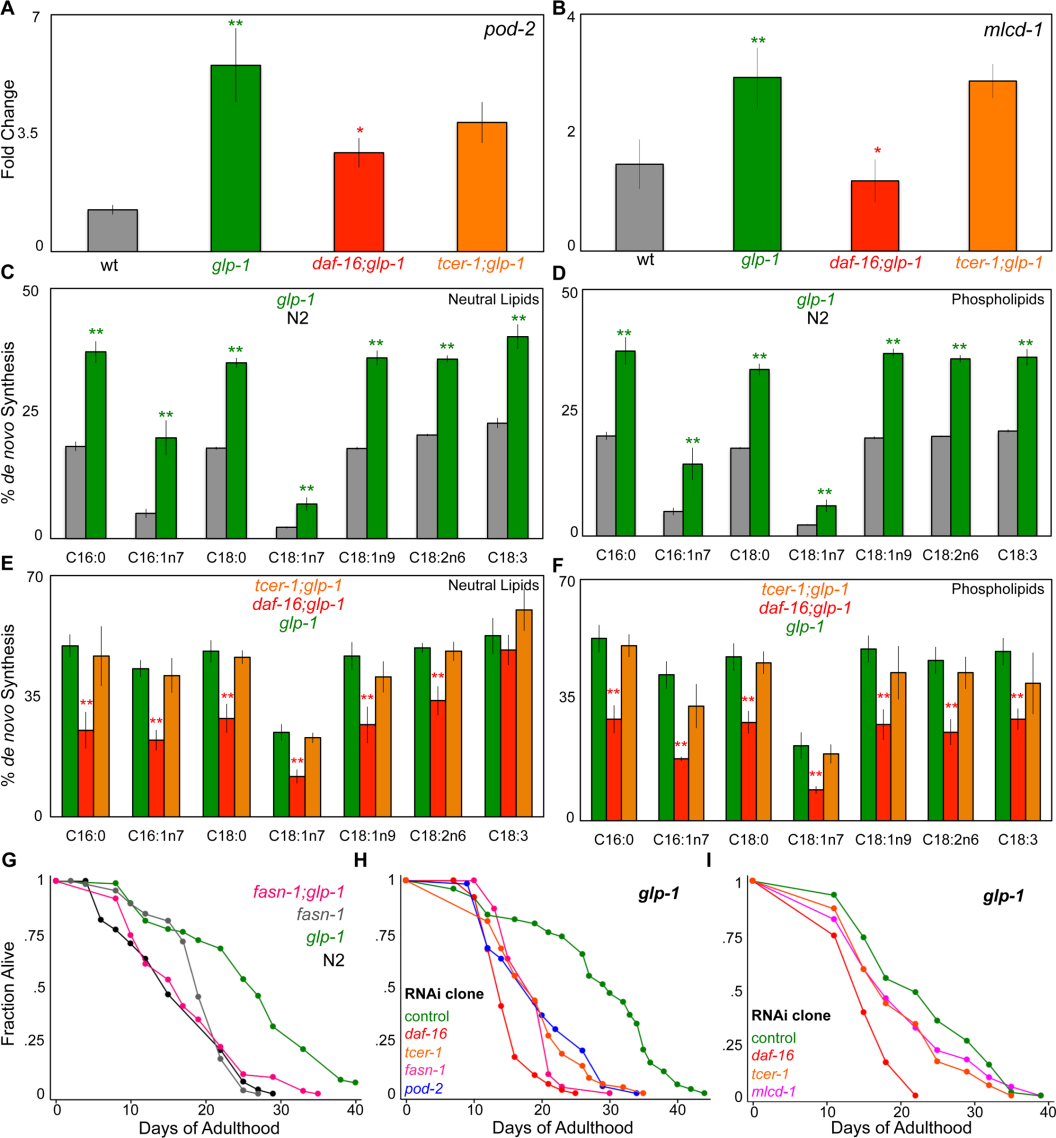
*de novo* fatty-acid synthesis is elevated following germline loss by DAF-16. **A, B.** DAF-16 mediates increased expression of *pod-2* and *mlcd-1* at least in part in germline-less adults. mRNA levels of *pod-2* (A) and *mlcd-1* (B) compared between wild-type (N2, gray), *glp-1* (green), *daf-16;glp-1* (red) and *tcer-1;glp-1* (orange) day 2 adults by Q-PCR. **C-F.** Germline loss causes increased *de novo* lipid synthesis in a *daf-16*-dependent manner. **C, D.** *de novo* fatty acid synthesis and dietary fat absorption and compared using a 13C isotope fatty-acid labeling assay [45] between late L4/early day 1 adults of wild type (N2, gray bars) and *glp-1* (green) strains. *de novo* lipid synthesis is significantly increased in both neutral lipid (C) and phospholipid (D) fractions of *glp-1* mutants. **E, F.** Day 2 adults of the sterile strains, *glp-1* (green), *daf-16;glp-1* (red) and *tcer-1;glp-1* (orange), were assessed through the same assay. In both neutral lipids (E) and phospholipids (F), *de novo* lipid synthesis is substantially reduced in *daf-16;glp-1* mutants, but not *tcer-1;glp-1* mutants, as predicted by RNA-Seq and Q-PCR. **G-I.** Knockdown of *de novo* fatty-acid synthesis genes impairs the longevity of *glp-1* adults. **G.** Effect of *fasn-1* mutation on the lifespan of *glp-1* mutants and wild-type (N2) worms. *glp-1* (green; m = 26.5 ± 0.5, n = 76/85), *fasn-1;glp-1* (pink; m = 18.0 ± 0.8, n = 84/88; P *vs*. *glp-1* <0.0001), N2 (black; m = 16.7 ± 0.7, n = 76/91), *fasn-1* (gray; m = 19.1 ± 0.6, n = 62/75; P *vs*. N2 <0.0001). **H, I.** Effect of RNAi knockdown of *pod-2*, *fasn-1* (H) and *mlcd-1* (I) on the lifespan of *glp-1* mutants. *glp-1* mutants were subjected to RNAi during adulthood by feeding bacteria containing empty control vector (green) as well as bacteria expressing dsRNA targeting different genes. In H: control (green; m = 27.9 ± 1.3, n = 49/50), *daf-16* (red; m = 14.8 ± 0.3, n = 74/78; P *vs*. control <0.0001), *tcer-1* (orange; m = 19.0 ± 0.6, n = 76/78; P *vs*. control <0.0001), *pod-2* (blue, m = 19.8 ± 0.8, n = 61/63, P *vs*. control, <0.0001) and *fasn-1* (pink, m = 18.5 ± 0.4, n = 69/72, P *vs*. control, <0.0001). In I: control (green; m = 23.3 ± 0.8, n = 77/91), *daf-16* (red; m = 15.7 ± 0.3, n = 83/110; P *vs*. control <0.0001), *tcer-1* (orange; m = 20.0 ± 0.7, n = 75/85; P *vs*. control <0.0001), *mlcd-1* (pink; m = 20.6 ± 1.1, n = 53/75, P *vs*. control, 0.05). In A-F, asterisks represent the statistical significance of differences observed in an unpaired, two-tailed t-test with P values 0.05 (*), 0.005 (**) or < 0.0005 (***). Green asterisks indicate the comparison between N2 and *glp-1*, whereas, red and orange asterisks depict the comparisons between *glp-1* and *daf-16;glp-1* or *tcer-1;glp-1*, respectively. Error bars represent the standard error of the mean. Data from additional trials is shown in S6 (G) and S7H and S7I Tables.

The first, and rate-limiting, step in the initiation of fatty-acid synthesis is mediated by the enzyme acetyl CoA carboxylase (ACC; encoded in *C*. *elegans* by *pod-2*) (Fig 2A) [42]. POD-2 catalyzes the synthesis of malonyl CoA (MCA), a critical intermediate that provides two-carbon units for the generation of the fatty acid palmitate (C16:0), the substrate for fatty-acid synthase (FASN-1, encoded by *fasn-1*). MCA levels are determined by the opposing activities of POD-2 and malonyl CoA decarboxylase 1(MLCD-1; encoded by *mlcd-1*) [42], an enzyme that converts it back to acetyl CoA [43, 44]. Both *pod-2* and *mlcd-1* were identified as DAF-16-Specific UP targets in our RNA-Seq data (S1B Table). Q-PCRs showed that the expression of both genes was enhanced in *glp-1* mutants and this upregulation was completely (*mlcd-1*) or partially (*pod-2*) impaired in *daf-16;glp-1* mutants (Fig 3A and 3B). *fasn-1*, although not picked up in the RNA-Seq dataset, showed a similar trend towards increased expression in *glp-1* mutants (S1A Fig). Based on these gene-expression changes we asked if germline ablation was accompanied by increased *de novo* fatty-acid synthesis. Using a 13C isotope fatty-acid labeling assay [45], we first compared *de novo* fatty-acid synthesis and absorption between wild-type worms and long-lived *glp-1* mutants. The eggs and embryos contained within the gonads of fertile adults confound lipid estimation hence this comparison could not be undertaken in day 2 adults; instead we used late L4/early Day 1 young adults before they became reproductively active. *glp-1* mutants exhibited a significant increase in *de novo* fatty-acid synthesis as compared to their fertile counterparts in both the neutral and phospholipid fractions (Fig 3C and 3D). To examine the contribution of DAF-16 and TCER-1 to this process, we compared *glp-1*, *daf-16;glp-1* and *tcer-1;glp-1* day 2 adults in the same assay. DAF-16 loss significantly reduced *de novo* fatty-acid synthesis and *tcer-1* did not have a major impact, in accordance with the RNA-Seq and Q-PCR data (Fig 3E and 3F). Next we addressed the functional relevance of this metabolic shift by examining the effect of inactivating these genes on *glp-1* mutants’ longevity. We introduced, *fasn-1(fr8)*, a partial loss-of-function allele of *fasn-1 [46],* into the *glp-1* background and found that it completely abrogated their longevity, whereas, *fasn-1* single mutant did not live significantly shorter than wild-type worms (Fig 3G and S6 Table). Adult-specific RNAi knockdown of *pod-2* and *mlcd-1* produced modest lifespan reductions (three of five trials and two of four trials, respectively; Fig 3H and 3I and S7 Table). *fasn-1* RNAi suppressed longevity consistently (Fig 3H and S7 Table). Together, these experiments revealed that germline loss triggers increased *de novo* fatty acid synthesis mediated by DAF-16, and implied that this enhancement is important, at least in part, for the subsequent lifespan extension.

### TAG production is enhanced in germline-less adults by DAF-16 and TCER-1

**Fig 4:**
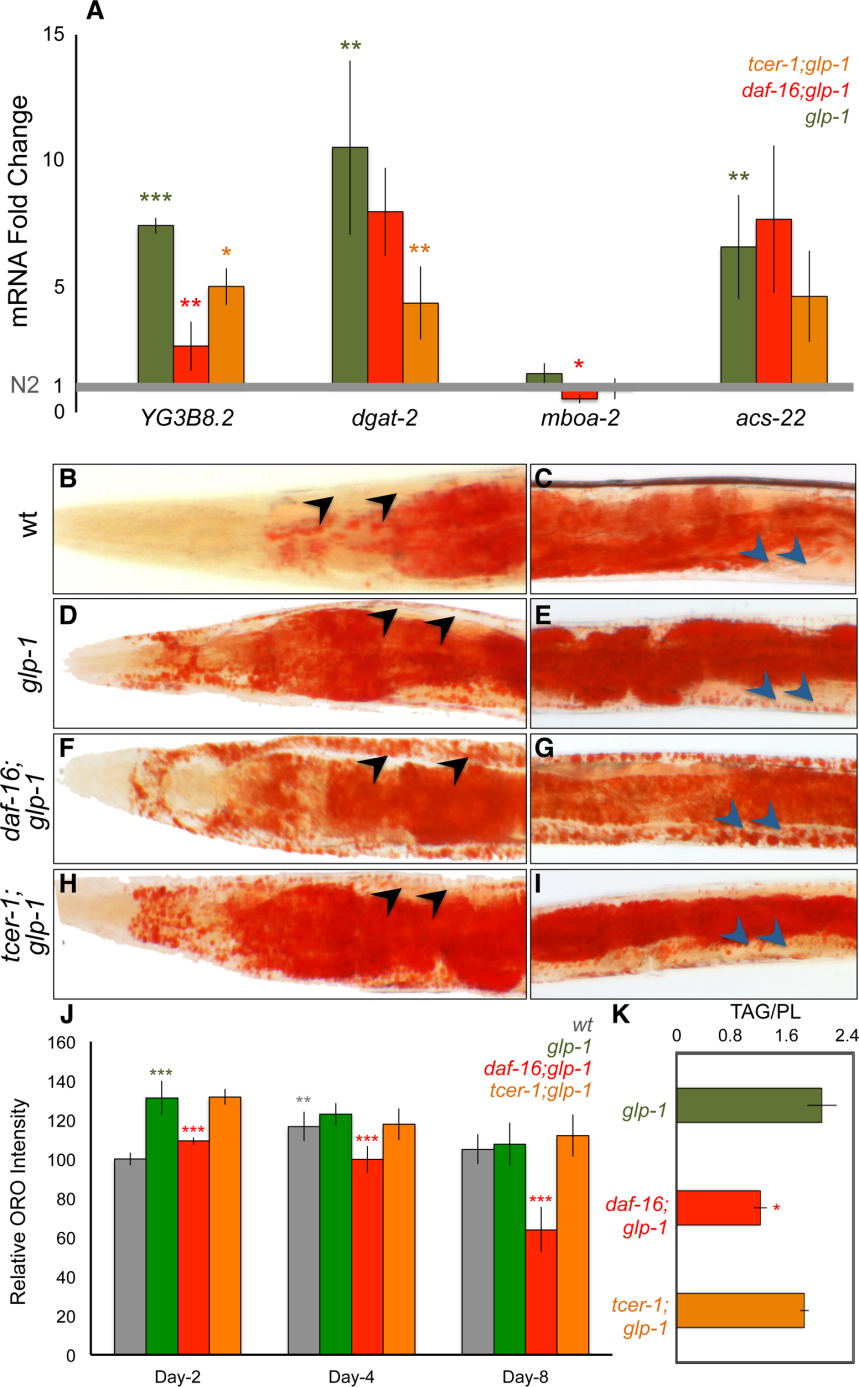
**A.** DAF-16 and TCER-1 mediate, in part, the increased expression of genes involved in triglyceride (TAG) production following germline removal. mRNA levels of genes encoding diacylglycerol acyl transferase (DGAT) enzymes that catalyze TAG production, compared relative to wild-type (N2, gray bar normalized to 1), between *glp-1* (green), *daf-16;glp-1* (red) and *tcer-1;glp-1* (orange) day 2 adults by Q-PCR. **B-J**. *daf-16;glp-1* mutants exhibit ectopic lipid deposition and undergo a progressive depletion of lipid stores with age. Lipid levels compared between different strains by ORO staining adults on days 2, 4 and 8 of adulthood. Representative images are shown in B-I and quantification is in J. The images focus on head region (B, D, F and H) and mid-body (C, E, G, I) of wild-type (N2, B, C) *glp-1* (D, E), *daf-16;glp-1* (F, G) and *tcer-1;glp-1* (H, I) day 6 adults. *daf-16;glp-1* mutants showed reduced ORO staining on day 2, as compared to *glp-1*. While N2 worms exhibit increased intestinal ORO after day 2, *daf-16;glp-1* mutants continue to undergo reduction in ORO levels. They also exhibit large, ectopic lipid droplets in muscle cells that are not observed in other strains (compare arrowheads between F, G and other panels). **J:** *daf-16;glp-1* mutants show decreased TAG levels.Using GC/MS, the triglyceride: phospholipid (TAG/PL) ratio of day 2 *daf-16;glp-1* adults was found to be significantly lesser than that of age-matched *glp-1* animals. *tcer-1;glp-1* mutants did not exhibit reduction in ORO staining nor biochemical TAG depletion, despite promoting the upregulation of DGAT genes. In A, J and K, asterisks represent the statistical significance of differences observed in an unpaired, two-tailed t-test with P values 0.05 (*), 0.005 (**) or < 0.0005 (***). Green asterisks indicate the comparison between N2 and *glp-1* whereas, red and orange asterisks depict the comparisons between *glp-1* and *daf-16;glp-1* or *tcer-1;glp-1*, respectively. The gray asterisks in J (day 4, N2 bar) show the comparison between N2 ORO levels on days 2 vs. 4. Error bars represent the standard error of the mean.

Fatty acids constitute the building blocks for the production of neutral lipids that are stored in the form of TAGs. The key step in this pathway is the conversion of DAGs into TAGs, catalyzed by the enzyme diacyl glycerol acyl transferase (DGAT) [47]. In *C*. *elegans*, six genes are predicted to encode DGATs [48]. Strikingly, four of these were identified in our RNA-Seq analysis as UP genes, being upregulated either by DAF-16 (*mboa-2*), TCER-1 (*dgat-2* and *acs-22*) or both (*Y53G8B*.*2*) (Fig 2 and S1 Table). Q-PCRs confirmed the upregulation of all four genes in *glp-1* mutants, and at least partially in a DAF-16- and/or TCER-1-dependent fashion (Fig 4A; *acs-22* expression was reduced in *tcer-1;glp-1* mutants, but did not achieve statistical significance). The fifth gene, *K07B1*.*4*, was identified in our previous study as a common target of DAF-16 and TCER-1 [24] (the sixth, *dgtr-1*, is predicted to be germline-restricted and thus would not have been detected in these experiments with germline-less mutants). Thus, the expression of a majority of the rate-limiting enzymes that catalyze the conversion of DAGs into TAGs was elevated upon germline loss by DAF-16 and/or TCER-1.

We next explored the biochemical ramifications of this increase in *‘dgat’* expression. First, fat content and distribution were examined using the lipid-labeling dye Oil Red O (ORO) [32]. In agreement with previous observations [32], young *glp-1* adults exhibited significantly higher levels of ORO staining than age-matched, wild type controls (Fig 4B–4E and 4J). We found that this increase was significantly attenuated in *daf-16;glp-1* mutants by day 2 of adulthood (Fig 4F and 4G and 4J), unlike a recent study that examined day 1 adults and reported an insignificant effect of the *daf-16* mutation on *glp-1* ORO levels [15]. We also discovered that the absence of DAF-16 caused a progressive depletion of intestinal fat with age. By day 8 of adulthood, ORO staining was markedly reduced in *daf-16;glp-1* mutants (Fig 4J). In addition, *daf-16;glp-1* adults exhibited ectopic fat deposition in other tissues such as body wall muscles (arrowheads in 4F, G). Surprisingly, *tcer-1;glp-1* mutants did not show any obvious changes in ORO staining or TAG levels (Fig 4H–4J). To verify the ORO observations, we used gas chromatography/mass spectrometry (GC/MS) and found TAG levels to be noticeably lower in *daf-16;glp-1* mutants, as compared to *glp-1* (Fig 4K). *tcer-1;glp-1* mutants, however, showed no significant difference from *glp-1* (Fig 4K). The reason for this discrepancy is unclear, but it is possible that this may reflect the partial loss of function nature of the *tcer-1(tm1452)* allele, unlike *daf-16(mu86)* which is a null.

**Fig 5:**
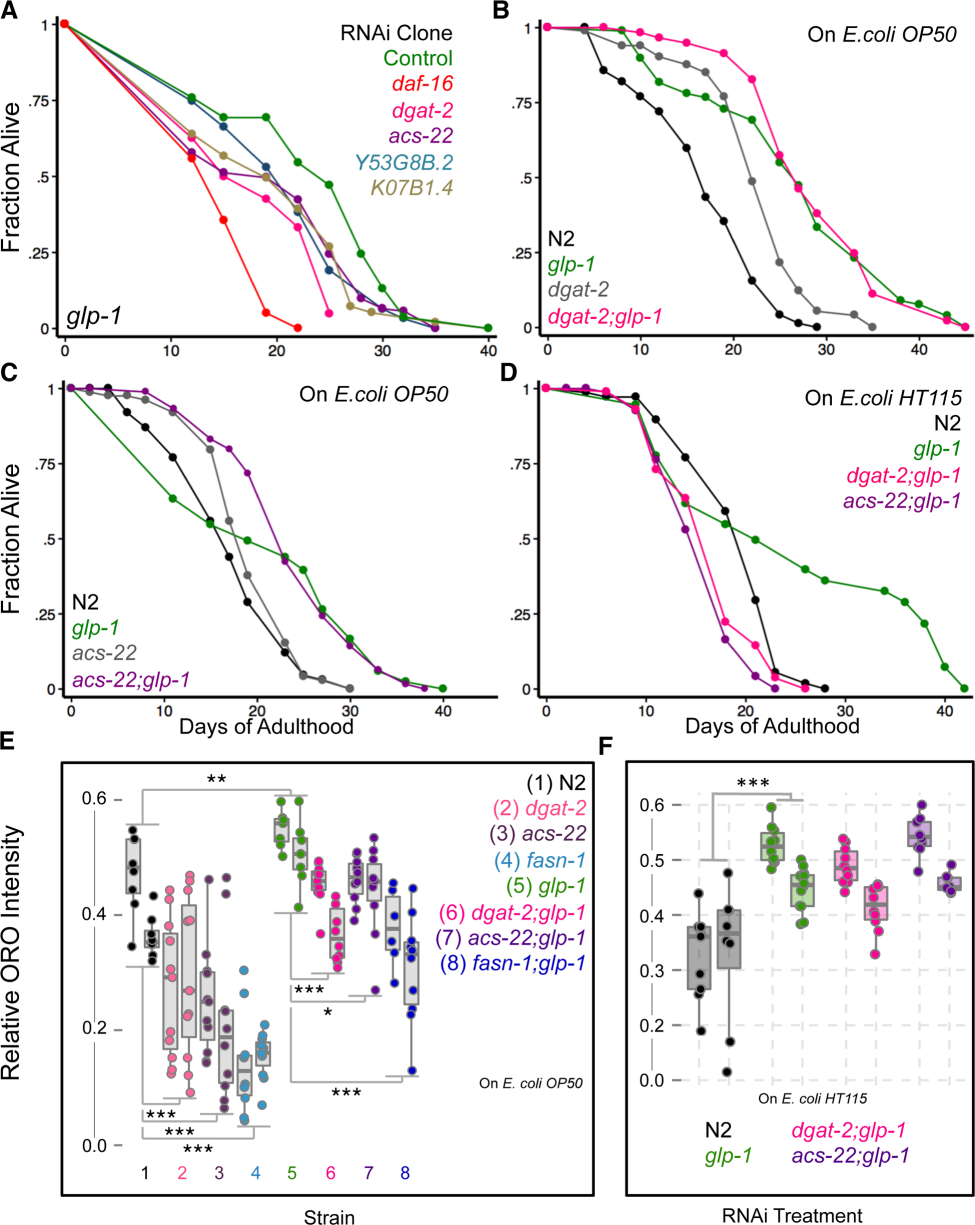
DGAT-2 and ACS-22 exhibit redundant and diet-dependent effects on the longevity and lipid content of germline-less mutants. **A.** RNAi inactivation ‘*dgat’* genes shortens *glp-1* mutants longevity modestly. *glp-1* mutants were subjected to RNAi during adulthood by feeding bacteria containing empty control vector (green) or bacteria expressing dsRNA targeting ‘*dgat’* genes. Control (green; m = 23.2 ± 0.3, n = 63/83), *daf-16* (red; m = 15.2 ± 0.3, n = 79/79; P *vs*. control <0.0001), *dgat-2* (pink; m = 18.5 ± 0.7, n = 71/80; P *vs*. control <0.0001), *acs-22* (purple, m = 19.7 ± 1.3, n = 123/123, P *vs*. control, 0.006), *Y53G8B*.*2* (blue, m = 20.7 ± 0.5, n = 59/71, P *vs*. control 0.02) and *K07B1*.*4* (sand, m = 19.9 ± 1.0, n = 97/97, P *vs*. control, <0.0001). **B, C.** Effect of *dgat-2(hj44)* and *acs-22(hj26)* mutations on the lifespan of *glp-1* mutants and wild-type (N2) worms on *E*. *coli OP50*. **B.** *glp-1* (green; m = 26.5 ± 0.5, n = 76/85), *dgat-2;glp-1* (pink; m = 28.5 ± 1.0, n = 52/61; P *vs*. *glp-1* 0.64), N2 (black; m = 16.7 ± 0.7, n = 76/91, P *vs*. *glp-1* <0.0001), *dgat-2* (gray; m = 22.6 ± 0.6, n = 75/88; P *vs*. N2 <0.0001). **C:** *glp-1* (green; m = 21.0 ± 0.9, n = 92/96), *acs-22;glp-1* (purple; m = 23.6 ± 0.6, n = 97/103; P *vs*. *glp-1* 0.87), N2 (black; m = 17.0 ± 0.7, n = 69/100), *acs-22* (gray; m = 19.1 ± 0.5, n = 72/83; P *vs*. N2 0.1). Additional trials are shown in S6B and S6C and S7(A) Tables. **D.** Effect of *dgat-2(hj44)* and *acs-22(hj26)* mutations on the lifespan of *glp-1* mutants grown during adulthood on *E*. *coli HT115* with the empty control vector. N2 (black; m = 19.16 ± 0.5, n = 57/75), *glp-1* (green; m = 24.2 ± 1.6, n = 43/60), *dgat-2;glp-1* (pink; m = 16.3 ± 0.4, n = 87/98; P *vs*. *glp-1* <0.0001), *acs-22;glp-1* (purple; m = 15.7 ± 0.4, n = 75/86). **E, F.** Effect of *dgat-2(hj44)*, *acs-22(hj26) and fasn-1(fr8)* mutations on fat levels of *glp-1* mutants. Quantification of lipid levels compared between different strains through ORO staining on day 2 adults grown on *E*. *coli OP50* (E) or *E*. *coli HT115* (F). The mutations caused reduced ORO staining in both fertile and *glp-1* backgrounds when grown on *E*. *coli OP50*. On *E*. *coli HT115*, *dgat-2;glp-1* and *acs-22;glp-1* did not show a significant ORO diminution. The box plots illustrate data from two biological replicates tested for each strain. Asterisks represent the P values {<0.05 (*), <0.005 (**) or <0.0001 (***)} derived using the *multcomp* package. See S6 Fig for ORO data of day 4 and 8 adults, and Methods for details of the statistical analyses.

To evaluate the functional significance of these gene-expression and biochemical changes, we asked if the ‘*dgat’* genes were essential for *glp-1* mutants longevity. RNAi inactivation of four of the *‘dgat’* genes that we tested caused modest reduction in *glp-1* mutants’ longevity but showed variability between trials. For instance, *dgat-2* and *Y53G8B*.*2* RNAi suppressed longevity significantly in three out of four trials, *K07B1*.*4* in two of three trials and *acs-22* in one of three trials (Fig 5A and S7 Table). We also introduced loss-of-function mutations of *dgat-2* and *acs-22* [48] into the *glp-1* background. Neither *dgat-2;glp-1* nor *acs-22;glp-1* lived shorter than *glp-1* mutants alone when fed the normal *E*. *coli OP50* diet (Fig 5B and 5C and S6 Table) although ORO staining was modestly reduced by both mutations (Fig 5D). Surprisingly, when these mutants were fed the *E*. *coli HT115* bacteria commonly used for RNAi experiments, lifespan extension *was* completely prevented (Fig 5E) but ORO levels remained the same (Fig 5F). The reasons for this discrepancy are unclear. It can possibly be explained by genetic redundancy between the *dgat-2* and *acs-22* in influencing germline-less longevity on *E*. *coli OP50* diet, but independent, essential functions on *E*. *coli HT115*. Overall, these experiments demonstrated that TAG production is enhanced in germline-less worms, at least partly, through DAF-16 and TCER-1-mediated increase in expression of *‘dgat’* genes, and these genes contribute at least modestly to germline-less longevity.

### DAF-16 and TCER-1 contribute to enhanced fatty-acid desaturation and elongation of germline-less adults

Previous studies [18], including ours [19], have shown that germline ablation is accompanied by increased expression of *‘fat’* genes encoding the fatty-acid desaturase enzymes that mediate conversion of saturated fatty acids (SFAs) to unsaturated fatty acids (UFAs) [49, 50]. In accordance with these reports, multiple *‘fat’* genes were identified in our RNA-Seq dataset (Fig 2 and S2A Fig). Our lipidomic analysis confirmed that *glp-1* mutants manifest increased UFAs in both the neutral and phospholipid fractions, as compared to fertile worms and this increase is attenuated in *daf-16*;*glp-1* mutants (S2D–S2K Fig). Fatty acid desaturation is closely linked to elongation of the carbon chains, mediated by elongase enzymes encoded by the *‘elo’* genes. Four out of nine *elo* genes encoded in the worm genome were picked up as DAF-16 and/or TCER-1 targets (Fig 2A and S2B and S2C Fig) and the abundance of long-chain (>C18) fatty acids was increased accordingly in *glp-1* mutants (S2D and S2E Fig) underscoring the importance of DAF-16 and TCER-1 in the increased abundance of UFAs and long-chain fatty acids in long-lived germline-ablated animals.

Altogether, our experiments collectively identified key genes required for initiation of *de novo* fat synthesis, fatty acid desaturation and elongation, and TAG production as being upregulated by DAF-16 and TCER-1 upon germline removal, and demonstrated the biochemical and functional relevance of these changes. They suggest that the enhancement of lipid anabolic processes is an important aspect of the response to germline removal and the ensuing lifespan enhancement.

### DAF-16 and TCER-1 upregulate the expression of genes involved in lipid catabolism in germline-less adults

**Fig 6:**
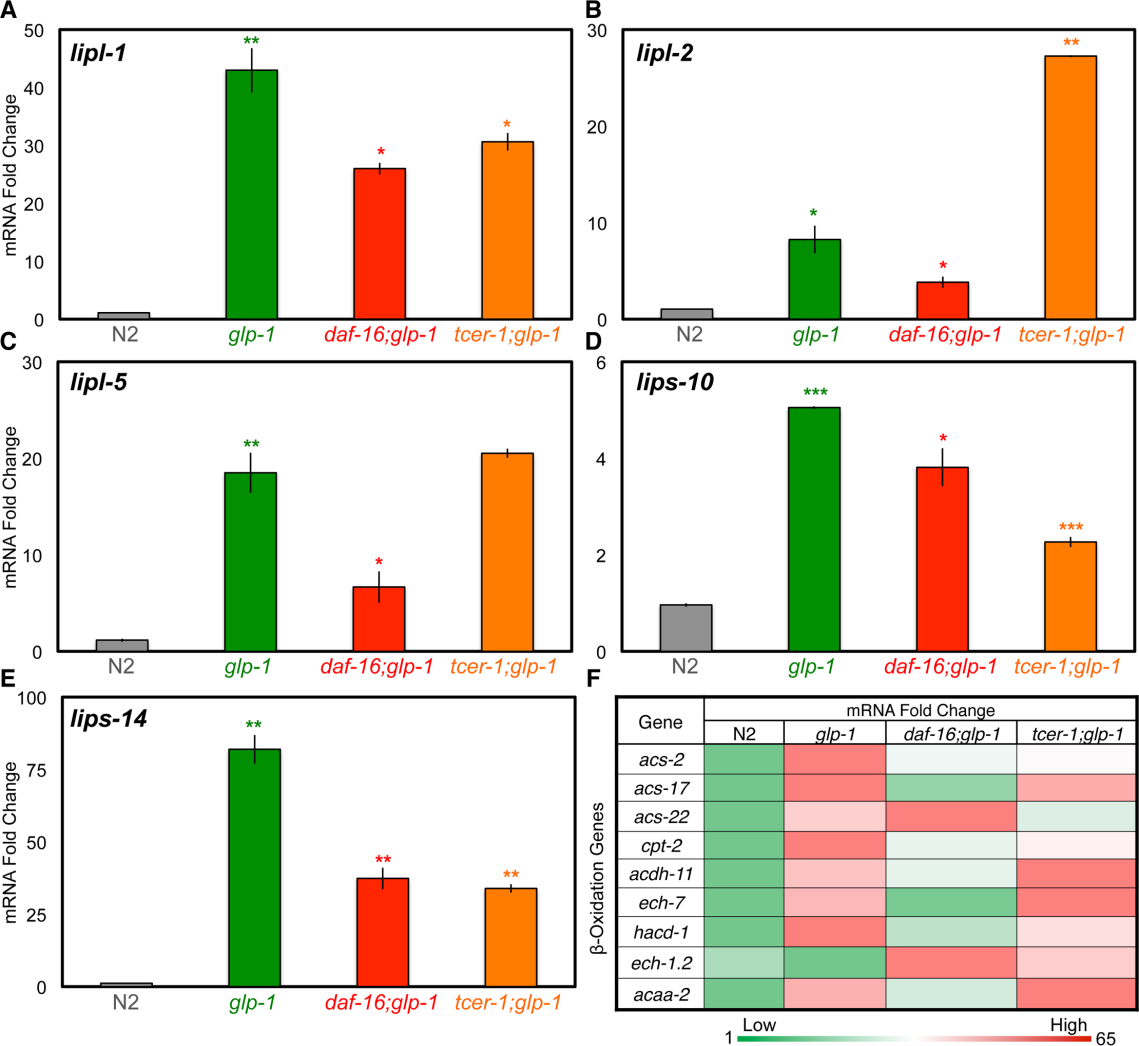
The expression of multiple lipases and fatty-acid β-oxidation genes is enhanced in germline-less adults through DAF-16 and TCER-1 activity. **A-E.** Expression of lipases and lipase-related genes in germline-less adults. mRNA levels of *lipl-1* (A), *lipl-2* (B), *lipl-5* (C), *lips-10* (D) and *lips-14* (E) compared between wild type (N2, gray), *glp-1* (green), *daf-16;glp-1* (red) and *tcer-1;glp-1* (orange) day 2 adults by Q-PCR. **F.** Expression of multiple mitochondrial β-oxidation genes is elevated in *glp-1* mutants, in part, by DAF-16, and by TCER-1. The heat map represents the combined results of Q-PCRs (conducted on the same stage as above) for genes predicted to function in mitochondrial β-oxidation. These genes were identified in RNA-Seq as UP genes and, as shown here, eight out of nine are increased in expression in *glp-1* mutants. The increased expression levels are attenuated for seven of eight genes in *daf-16;glp-1* mutants and for four of eight genes in *tcer-1;glp-1* mutants. The color code is shown at the bottom of the panel. Green represents low levels of expression and red higher. Highest and smallest fold change values are indicated at each end of the color bar. In A-E, asterisks represent the statistical significance of the differences observed in an unpaired, two-tailed t-test with P values 0.05 (*), 0.005 (**) or < 0.0005 (***). Green asterisks indicate the comparison between N2 and *glp-1* whereas, red and orange asterisks depict the comparisons between *glp-1* and *daf-16;glp-1* or *tcer-1;glp-1*, respectively. Error bars denote the standard error of the mean.

Lipid breakdown is initiated by the hydrolysis of TAGs by lipases to produce DAGs and free fatty acids (FFAs). Six lipases were identified as UP targets in our RNA-Seq analysis. In Q-PCR assays, five of these exhibited five-to-eighty fold increase in expression in *glp-1* mutants in a partially *daf-16*-dependent manner (Fig 6A–6E). We were unable to confirm the RNA-Seq data for the sixth lipase, *atgl-1/*ATGL-1 (S1B Fig). Each of the five lipases we identified was essential for *glp-1* mutants’ longevity, including *lipl-1* and *lipl-2* that were predicted to be upregulated by DAF-16 but downregulated by TCER-1 (Fig 2 and S1H Table). Q-PCRs showed that *lipl-1* was in fact partially upregulated by both factors and only *lipl-2* was repressed by TCER-1; however, RNAi inactivation of both genes caused similar suppression of lifespan. The identification of multiple lipases is in keeping with a previous report that found *glp-1* mutants display increased lipase activity partially dependent on DAF-16 and the lipase, LIPL-4 [13]. Taken together these data suggest that lipolysis may be broadly enhanced upon germline loss.

The FFAs released upon TAG hydrolysis are broken down further to produce acetyl Co A through fatty-acid β-oxidation (Fig 2B). Eighteen peroxisomal- and mitochondrial-β-oxidation genes [51] were represented in our RNA-Seq dataset (thirteen UP, three DOWN and two with opposite effects), in keeping with our recent demonstration that the expression of multiple mitochondrial β-oxidation genes is enhanced in *glp-1* mutants through NHR-49 activity [19]. Q-PCRs confirmed that the upregulation of these genes in *glp-1* mutants was partially suppressed by *daf-16* and/or *tcer-1* mutations as well (Fig 6F). *nhr-49* was included in the Joint UP class, as predicted by our previous study [19]. This identification of these lipases and β-oxidation genes, along with the evidence above that multiple lipogenic processes were augmented, suggest that germline loss may trigger a simultaneous increase in lipid production and breakdown, although this remains to be demonstrated directly (see Discussion).

### DAF-16 and TCER-1 repress protein synthesis and reproduction-related gene expression following germline loss

The physiology of fertile, young adults is highly invested in macromolecular synthesis to support growth and reproduction. Loss of the germline induces a fundamental change in this metabolic state, and an inability to suppress the growth programs already in place can be detrimental to the animal. Thus, genes downregulated by DAF-16 and TCER-1 following germline ablation are likely to be as important as those that are upregulated. With this perspective, we examined the DOWN genes and found them to be highly enriched for molecular processes associated with active procreation such as protein translation and reproduction (Fig 1B and S3A–S3C Fig).

DAVID analysis of the DAF-16-Specific DOWN genes revealed protein synthesis as the predominant category (Fig 1B). One of the main GO categories (Enrichment Score 3.08) included eighteen genes encoding proteins involved in translation subunits {nine large and seven small ribosomal protein (RP) subunits}, a translation initiation factor, EIF-6, and the small mitochondrial ribosomal protein subunit (MRPS), MRPS-23 (S3A Fig and S4D Table). In addition, expression of three tRNA synthetase genes was repressed by DAF-16 (S4D Table). Since protein synthesis is a key requirement for a proliferating germline, it is plausible that germline removal triggers DAF-16-dependent repression of translation.

DAVID analysis of TCER-1-Specific DOWN class showed that, within the group receiving the highest enrichment score (2.3), 12/53 genes encoded proteins involved in splicing or RNA processing (Table 1 and S4G and S4H Table). This is in accordance with the role of human TCERG1 in regulating elongation-associated splicing [52]. A detailed examination of the genes in this group also revealed that the knock down of 37/53 genes has been reported to result in reproductive phenotypes including gonadal defects, reduced brood size and sterility (Table 1). Reproduction was also one of the highly represented groups in the REVIGO analysis (S5D Table). These observations implied that once the germline is lost, TCER-1 actively repressed the somatic program of reproduction. Thus, DAF-16 and TCER-1 may together facilitate the adaptation to germline removal by terminating the gene-expression programs that support reproductive physiology in the somatic cells of the animal.

**Table 1:** Genes repressed by TCER-1 following germline loss are predominantly required for optimal fertility in normal animals. DAVID analysis and Gene Functional Classification of TCER-1-Specific DOWN revealed 53 genes in the group with the highest enrichment score (2.3). These are listed here along with their molecular identities and known human orthologs. See S4 Table for details of DAVID analyses and S5 Table for supporting data from REVIGO analysis. Many of these genes function in splicing and RNA processing (highlighted in bold). Strikingly, inactivation of 37/53 genes has been reported to elicit reproductive phenotypes enumerated here (70%, p<0.0001).

TCER-1-Specific DOWN Gene Group 1 (Enrichment Score: 2.3)			
Gene (Cosmid)	Molecular Function	Human Ortholog	Reproductive Defects Associated with Gene Reduction-of-Function
***uaf-2 (Y92C3B*.*2)***	Splicing factor U2AF, RNA-binding protein	U2AF1: Small nuclear ribonucleoprotein auxiiary splicing factor	Sterility, multiple gonadal, vulval and germ-cell defects [79,81,87]
***prp-6 (Y59A8B*.*6)***	Yeast PRP (splicing factor)-related	XAB2: Pre-mRNA-splicing factor SYF1; PRPF6: Pre-mRNA-processing splicing factor 6	Sterility, DTC defects [76,77]
***rsp-7 (D2089*.*1)***	Member of SR family of nuclear phosphoprotein splicing factors	SRSF11: Ser/Arg-rich splicing factor 11; SREK1: Glu/Lys-rich splicing regulatory protein 1	Sterility^
***rnp-6 (Y47G6A*.*20)***	RNP (RRM RNA binding) domain-containing protein; predicted to function in splicing	PUF60: Poly(U)-binding-splicing factor	Sterility, multiple vulval and germ-cell defects [76,85]
***pcf-11 (R144*.*2)***	Cleavage and polyadenylation factor	PCF11: Pre-mRNA cleavage complex 2 protein	Sterility, multiple germ-cell defects [76,81]
***hel-1 (C26D10*.*2)***	Helicase	UAP56: Splicosome assembly protein; DDX39B: Spliceosome RNA helicase	Sterility, multiple vulval and germ-cell defects [79]
***tiar-1 (C18A3*.*5)***	TIA1/TIAL RNA binding protein	TIAL1: TIA1 granule associated RNA-binding protein	Reduced brood size, sterility, vulval defects [76,83]
***asd-2 (T21G5*.*5)***	KH-domain protein involved in splicing and mRNA-export	QK1: KH domain RNA-binding and splicing protein, Quaking	Reduced brood size, vulval and egg-laying defects [76]
***ddx-15 (F56D2*.*6)***	DEAH helicase, putative pre-mRNA-splicing factor	DHX15: Putative pre-mRNA-splicing factor ATP-dependent RNA helicase	Gonad and germline morphology defects, sterility [76,85]
***lst-3 (Y37A1B*.*1)***	RNA-binding domain	CCAR1: Cell division and apoptosis regulator protein 1	Reduced brood size, vulval defects [90]^
***F34D10*.*4***	Shared homology with human putative RNA-binding protein		
***F09F7*.*5***	Alternatively spliced protein that in conjunction with LIN-35 and CLK-1 maintains large brood size and short lifespan, respectively		Reduced brood size [76]
*osm-7 (T05D4*.*4)*	Novel protein required for osmosensation		Maternal sterile, vulval defects [85]
*lin-14 (T25C12.*1)*	Novel protein involved in developmental timing		Gonad and vulval morphology defects, egg-laying phentoypes, sterile progeny, long lifespan [75,87]
*nuo-3 (Y57G11C.*12)*	Mitochondrial NADH ubiquinone oxidoreductase	NDUFA6: NADH dehydrogenase (ubiquinone) 1 alpha subcomplex subunit 6	Sterility, long lifespan [17,80]
*Y39B6A.*3*	Mitochondrial Fe/S assembly protein	ISCA1: Iron-sulfur cluster assembly 1	long lifespan [86]
*mrpl-17 (Y54E10A*.*7)*	Mitochondria-encoded large ribosomal protein	MRPL17: mitochondrial 39S ribosomal protein L17	Sterility^
*mrps-5 (E02A10.*1*	Mitochondria-encoded small ribosomal protein	MRPS5: 28S mitochondrial ribosomal protein S5	Reduced brood size, long lifespan [55,85]
*sams-3 (C06E7.*1)*	S-adenosyl methionine synthatase	MAT2A, MAT1A: S-adenosylmethionine synthases	Reduced brood size, sterility, vulval phenotypes, long lifespan [76,78,86]
*sams-4 (C06E7*.*3)*	S-adenosylmethionine synthetase	MAT2A, MAT1A: S-adenosylmethionine synthases	Vulval defects, sterile progeny [87]
*phi-58 (T07A9*.*9)*	Probable nucleolar GTPase involved in 60S ribosome synthesis	GTPB4: Nucleolar GTP-binding protein 1	Multiple gonadal and germ cell defects, reduced brood size, sterility [80,85]
*cars-1 (Y23H5A*.*7)*	Cysteinyl tRNA synthetase	CARS: Cysteinyl tRNA synthetase	Sterility, gonadal and germ-cell defects [79,89]
*rnh-1*.*0 (F59A6*.*6)*	Putative mitochondrial RNAse H	RNASEH1: Ribonuclease H1	
*prmt-1 (Y113G7B*.*17)*	Protein arginine methyl transferase	PRMT1 and 8, HRMT1L2: protein arginine N-methyltransferase	Reduced brood size, vulval defects [85,87]
*elo-5 (F41H10*.*7)*	Polyunsaturated fatty acid (PUFA) elongase	ELOVL3 (Elongation of very long chain fatty acids)	
*E04F6*.*4*	Phospholipase	PLD4 and PLD5 (Phospholipases)	Reduced brood size, sterility [76]
*oac-14 (F09B9*.*1)*	O-acyl transferase		
*C23H3*.*3*		ASCC1: Activating Signal CoIntegrator Complex 1	
*npp-9 (F59A2*.*1)*	Nuclear pore complex protein	RANBP2: Sumo E3 ligase	Gonadal, vulval and germ-cel -defects, sterility, reduced brood size [77,80,85]
*Y17G7B*.*20*		CEBPs: CCAAT/enhancer-binding proteins	Sterile progeny [82]
*trpp-11 (B0412*.*3)*	Transport protein particle	TRAPPC11: Trafficking protein complex subunit 11	Reduced brood size, sterility [84]^
*syx-16 (ZC155*.*7)*	vessicle traffic protein syntaxin	STX16: Syntaxin 16	
*rme-8 (F18C12*.*2)*	DNAJ domain containing protein	DNAJC13, DnaJ homolog subfamily C member 13	Reduced brood size, germ cell defects [79]
*F10G7*.*5*		Monocarboxylate transporter 14	
*F27C1*.*2*	Putative Cu transporter	SLC31A1: high affinity copper uptake protein 1	Maternal sterile [85,87]
*C42D4*.*2*		CEL: bile salt-activated lipase precursor Carboxylesterase 1 precursor	
*dnj-29 (Y63D3A*.*6)*	DNaJ domain protein	SEC63: Translocation protein	Sterile^
*gyg-1 (F56B6*.*4)*	Glycogenin like protein	GYG1: Glycogenin1	Reduced brood size, sterile progeny [85]
*vab-10 (ZK1151*.*1)*	Spectraplakin	DST: Dystonin, MACF1	Somatic gonadal, vulval defects and germ cell defects, reduced brood size, sterility [80,88]
*C53H9*.*2*	Likely GTPase	LSG1: Large subunit GTPase	Sterility, vulval defects [85,89]
*wapl-1 (R08C7*.*10)*	*Drosophila* Wings APart-Like cohesin interactor	WAPAL: Isoform of Wings apart-like protein	Sterile^
*pqn-92 (Y75B8A*.*27)*	Prion-like-(Q/N-rich)- domain-bearing protein		
*clr-1 (F56D1*.*4)*	Receptor tyrosine phosphatase in FGF pathway	PTPR: Receptor-type tyrosine-protein phosphatase	Reduced brood size, vulval defects, sterile progeny [85,87]
*ifo-1 (F42C5*.*10)*	Intermediate filament organizer		
*rig-6 (C33F10*.*5)*	NeuRonal IGCAM with immunoglobulin domain	CNTN2: Contactin	Germ cell defects, maternal sterile [79,82]
*let-805 (H19M22*.*2)*	Myotactin	FN1: Fibronectin	Maternal sterile [81]
*phi-59 (T19B10*.*2)*			Reduced brood size, sterility, vulval defects [76,87]
*C15C7*.*5*		Uncharacterized protein	
*Y19D10A*.*16*	Aldose 1 Epimerase	GALM: Aldose 1 epimerase	
*C01B4*.*6*	Aldose 1 Epimerase	GALM: Aldose 1 epimerase	
*Y57E12AL*.*1*		SERINC1: Serine incorporator 1	
*F56C9*.*7*	Protein with domain of unknown function (DUF)		
*ZC247*.*1*		Uncharacterized protein	Germ cell defects, maternal sterile [79,82]

^ Wormbase curation reports reproductive defects in mutants generated by knock-out consortia (www.wormbase.org)

*Gene inactivation increases lifespan of normal adults

### TCER-1 represses the expression of anti-longevity genes in *glp-1* mutants

The genes most repressed by TCER-1 also included several factors whose reductions-of-function have been reported to increase the lifespan of normal adults (highlighted in bold in Table 1). Indeed, ‘Aging and Adult Lifespan’ was one of the main groups that emerged from the DAVID analysis of TCER-1 DOWN class (Fig 1B). This led us to ask if TCER-1 inhibited the expression of anti-longevity genes. Since the DOWN genes were predicted to be already repressed in germline-less animals, testing their anti-longevity roles in *glp-1* mutants was not feasible. Hence, we examined their function in wild-type animals. We used a *fer-15;fem-1* temperature-sensitive mutant strain that is sterile when grown at the non-permissive temperature but exhibits normal lifespan and has been used extensively as a surrogate for wild-type worms in large-scale lifespan analyses [16, 17]. We asked if inactivation of TCER-1 DOWN genes had a beneficial effect on the lifespan of *fer-15;fem-1* adults. To circumvent developmental requirements, RNAi was initiated on day 1 of adulthood. Upon RNAi inactivation of nineteen of the most highly repressed TCER-1-Specific DOWN genes, we observed a statistically significant lifespan extension in thirteen cases, eight of which were reproduced in at least two trials (Fig 1C and S8 Table). To substantiate the RNAi data, we also tested the lifespans of six strains carrying mutations in five of these genes. Two mutants, *gst-24* and *dopy-6*, showed consistent and statistically significant lifespan extensions compared to wild-type worms. Two others (*numr-1* and *lys-4*) showed significant but variable lifespan increments (S9 Table; unlike the RNAi strategy, adult-specific gene knockdown was not possible with mutants and sickness resulting from developmental defects could not be avoided, as was the case with *lin-17*). These data lead us to posit that TCER-1’s repressor functions following germline removal may include suppressing aspects of reproductive physiology, by inhibiting the expression of reproductive genes, as well as the expression of anti-longevity genes.

### *tcer-1* is essential for the reproductive health of fertile animals

**Fig 7:**
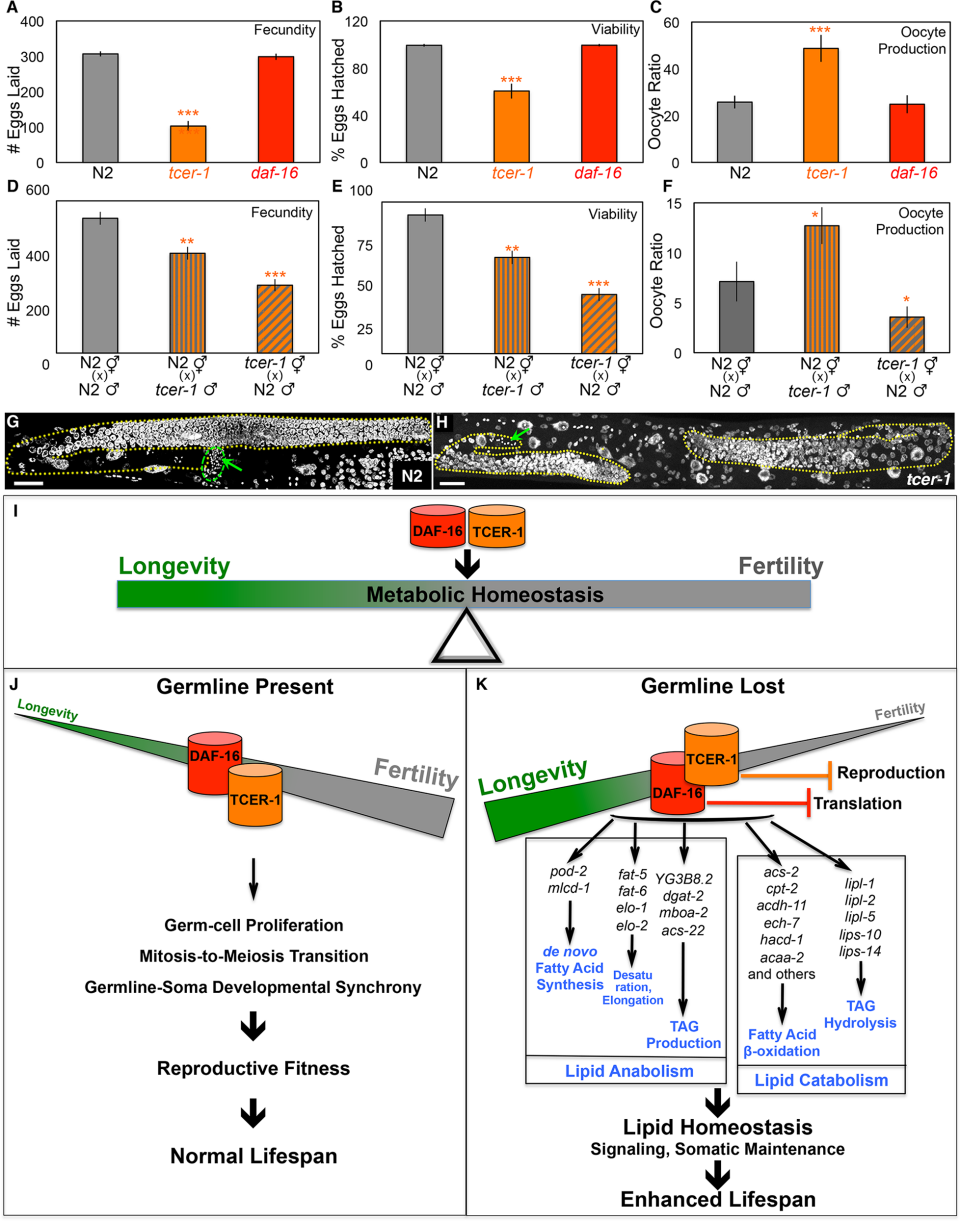
TCER-1 promotes reproductive health in normal, fertile worms and metabolic homeostasis and longevity in germline-less animals. **A-C.** *tcer-1* mutants exhibit multiple fertility defects. Reproductive fitness parameters compared between wild type (N2, gray), *daf-16* (red) and *tcer-1* (orange) mutants, including fecundity (number of eggs laid, A), viability (percent of laid eggs that hatch successfully, B), oocyte production (C) grown at 20°C. *tcer-1*, but not *daf-16*, mutants are defective in all aspects and these phenotypes are aggravated further at 25°C (S4C Fig). **D-F.** *tcer-1* mutants make defective sperm and oocytes. Functionality of *tcer-1* mutants’ sperm and oocytes was assessed by crossing *tcer-1* males to wild-type (N2) hermaphrodites (vertical striped bars) and *tcer-1* hermaphrodites to N2 males (diagonal striped bars), respectively. Reproductive health measures were compared against control N2 hermaphrodites and males crossed to each other (gray bars). Shown here are results for fecundity (D) viability of eggs (E) and oocyte production (F). **G, H.** *tcer-1* mutants exhibit inadequate germ-cell development and germline heterochrony. DAPI stained germline of wild type (N2) day 1 adults germ cell nuclei organized in spatial-temporal gradient, whereas, gonads from *tcer-1* mutants exhibit significantly reduced germ-cell number. Gonads are outlined with yellow, dotted lines and scale bar in both images is 20μM. Germline heterochrony in the mutants is revealed by the presence of sperm (green arrows) within the proliferating region of the gonad and not within the spermatheca (demarcated with a green dotted line) as seen in N2. Quantification of germline defects is shown in S4 Fig. In A-F, asterisks represent the statistical significance of differences observed in an unpaired, two-tailed t-test with P values 0.05 (*), 0.005 (**) or < 0.0005 (***) between N2 and *tcer-1* (orange asterisks) or *daf-16* (red asterisks). **I-K.** Schematic representation of the model proposed based on the findings of this study. Our results suggest mechanisms by which DAF-16 and TCER-1 help maintain metabolic homeostasis in response to changes in the reproductive status of the animal (I). In fertile animals, TCER-1 ensures reproductive fitness, likely by promoting germ-cell proliferation and successful sperm-to-oocyte production switch in synchrony with somatic development (J). When the germline is lost, as in the case of *glp-1* mutants, TCER-1 undergoes a role-reversal and inhibits the somatic program of reproduction instead (K). TCER-1 also represses anti-longevity genes independently. DAF-16, on the other hand restrains translation by repressing ribosomal-gene expression (K). Additionally, DAF-16 and TCER-1 enhance the expression of genes involved in both lipid-anabolic and lipid-catabolic pathways (depicted in K) in adaptation to germline depletion. We posit that the simultaneous enhancement of these ostensibly antagonistic processes (a) allows the animal to retain metabolic homeostasis during the physiological flux caused by germline loss, and (b) enables the production of lipid signaling moieties and/or ligands for transcription factors whose activities ultimately advance longevity.

The human TCERG1 gene is highly expressed in oocytes where its mRNA levels decline with age [53]. These reports, together with our observations that genes with reproductive roles are enriched in the TCER-1-Specific DOWN class, and that *tcer-1* mutants produced fewer progeny than wild-type animals, prompted us to investigate *tcer-1*’s function in reproduction in normal, fertile worms. We employed a collection of assays to assess reproductive health, including quantifying the brood size (total number of eggs laid), viability (fraction of eggs that hatch successfully) and reproductive span (the duration of adulthood for which reproduction is maintained) [54]. As worms age, they begin to lay unfertilized oocytes. We quantified oocyte production to obtain another measure of reproductive health, ‘oocyte ratio’ (the ratio of total number of eggs laid to the number of oocytes laid) (see Methods for details). We found that at normal growth temperature (20°C), *tcer-1* mutants laid ~65% fewer eggs than wild-type worms (Fig 7A) and the hatching rate of these eggs was ~40% less than that of wild type (Fig 7B). The oocyte ratio was also significantly increased (Fig 7C). The mutants started laying oocytes earlier than wild type and continued to do so late into middle age (S4A Fig). In contrast, *daf-16* mutants did not exhibit any of these phenotypes (Fig 7A–7C and S4A Fig). To identify whether *tcer-1* mutants’ fertility phenotypes were due to defects in sperm, oocytes (or both), we repeated these reproductive health assays on reciprocal crosses: wild type hermaphrodites mated to *tcer-1* mutant males (to assess sperm health) or *tcer-1* mutant hermaphrodites mated to wild type males (to assess oocytes). In both cases, we observed reduced brood size and decreased viability (Fig 7D and 7E) and oocyte abnormalities (Fig 7F and S4B Fig) indicating that TCER-1 is necessary in both gametes for fertility, although this does not obviate the role of somatic TCER-1 in fertility.

### *tcer-1* mutants display germline heterochrony and germ cell defects

The *tcer-1* reproductive phenotypes suggested that there might be underlying defects in germ cell development or meiosis. We explored this by examining the germline for the number of DAPI-staining bodies at diakinesis, a read-out for meiotic crossover formation. In both wild type and *tcer-1* mutant animals, six DAPI-staining bodies were noticeable in all the oocytes imaged indicating that crossovers were properly made between all the chromosomal homolog pairs. However, we noticed that ~30% of day 1 *tcer-1* mutant adults were still undergoing spermatogenesis (evidenced by presence of sperm in the gonadal arm rather than being restricted to the spermatheca), which is normally completed during the early L4 larval stage (Fig 7G and 7H). This indicated a defect in the developmental switch from spermatogenesis to oogenesis. To determine whether the animals failed to switch completely or were delayed in switching, we analyzed older adults. By day two, oogenesis was noticeable in all the animals (S4C Fig). We interpret this data to suggest that *tcer-1* mutants exhibit germline heterochrony or a delay in switching from spermatogenesis to oogenesis in sync with somatic maturation. These defects were further aggravated when the worms were grown at higher temperatures. By the second generation at 25°C, *tcer-1* mutants showed a precipitous decline in fecundity accompanied by dramatic changes in germline morphology; >70% had germline defects including germline heterochrony, small germlines with few-to-no germ cells as well as reduced sperm count, despite prolonged spermatogenesis (S4C–S4E Fig). Together this spectrum of defects revealed that *tcer-1* is required for normal germ-cell growth, differentiation and germline-soma synchrony, the latter being most sensitive to loss of *tcer-1* function. Taken together, our results indicate the *tcer-1* promotes reproductive health in normal, fertile adults, but upon germline loss, it switches roles to repress reproductive physiology and promote the expression of genes that ensure metabolic homeostasis and longevity (Fig 7I–7K).

## Discussion

Changes in the reproductive state- be it pubertal maturation, menopause or sudden loss of fertility- can be challenging to an animal’s physiology. How does an organism maintain metabolic homeostasis while transforming its molecular and energetic profile to match its procreative status? We addressed this topic by exploring the GRNs governed by DAF-16 and TCER-1 in germline-less *C*. *elegans* mutants that exhibit improved health and lifespan. We discovered that the expression of both lipid synthesis and degradation genes is enhanced by DAF-16 and TCER-1 in response to germline removal. Our data suggest that the coordinated augmentation of these processes may enable the preservation of metabolic homeostasis and facilitate adaptation to germline loss. We also identified repressive functions of DAF-16 and TCER-1 that help impede gene-expression programs intended to support procreation. Finally, in an unexpected discovery, we showed that TCER-1 promotes fertility in normal animals, indicating a role for the protein in maintaining the balance between fertility and longevity (Fig 7J and 7K).

### The GRNs governed by DAF-16 and TCER-1 upon germline loss

RNA-Seq analysis allowed us to describe a detailed picture of the GRNs governed by DAF-16 and TCER-1 following germline loss, including the first compilation of TCER-1 downstream genes. Our Q-PCR and lifespan assays indicate that many of the DAF-16 and/or TCER-1 UP targets are genes whose expression is, at least partially, increased following germline depletion, and which promote GSC-less longevity. A majority these genes’ inactivations caused moderate lifespan reduction (~10 to 20%). While it cannot be ruled out that these modest effects are due to incomplete knockdown caused by RNAi, it is also possible that some UP genes make incremental individual contributions, and the collective result is a substantial life lengthening. Interestingly, the UP group included several transcription factors, some reported to be essential for *glp-1* mutants’ longevity (NHR-49, HLH-30, DAF-12, SKN-1 and MDT-15) [4, 14–16, 19, 37] (highlighted in S1 Table) and new ones that implicate novel cellular processes in this longevity pathway. For instance, *atfs-1*, a key mediator of mitochondrial unfolded protein response (mitoUPR), was included in the TCER-1-Specific UP group and was essential for *glp-1* longevity (S3 Table). Induction of mitoUPR by knockdown of MRPSs increases lifespan in worms and mice [55]. Notably, we identified multiple MRPS-encoding genes as being repressed by DAF-16 and/or TCER-1 (Table 1 and S3 Fig). These observations raise the enticing possibility that GSC loss may also activate mitoUPR. Previously we showed that DAF-16 and TCER-1 partly mediated *nhr-49* upregulation in *glp-1* mutants. Multiple β-oxidation genes that we had found to be upregulated by NHR-49 in *glp-1* mutants [19] were represented in the RNA-Seq dataset, along with *nhr-49* itself. However, we noticed that the *daf-16* mutation had a modest effect on the mitochondrial β-oxidation genes as compared to *nhr-49*. One possible interpretation of this data is that DAF-16 and TCER-1’s predominant role may be upregulating key transcription factors that in turn activate specific cellular processes. Accordingly, two HLH-30-regulated autophagy genes, *bec-1* and *atg-9*, were included in the UP class. However, the activities of many of these factors are controlled at post-translational steps such as nuclear localization (SKN-1, HLH-30) [14, 15] and we did not observe a strong or consistent influence of DAF-16 or TCER-1 on their expression using Q-PCRs (S5 Fig), so the significance of these factors being identified in the RNA-Seq dataset remains unclear. The data underscore the complexity of the GRNs activated by GSC removal and suggest new relationships between previously known genes important for *glp-1* mutants’ longevity.

### The activation of lipogenic pathways in response to germline loss

We found that multiple lipogenic genes were upregulated by DAF-16 and TCER-1 in *glp-1* mutants, and that at least three lipid anabolic processes- *de novo* fatty-acid synthesis, TAG production and fatty-acid desaturation and elongation- were all significantly elevated upon GSC removal. The identification of increased *de novo* fatty acid synthesis was particularly intriguing, because recent evidence suggests that a large fraction of the surplus fat in *glp-1* mutants is likely to be a result of unchecked yolk production [15]. Yolk lipids are similar to *bonafide* storage forms of fat but they also have distinct biochemical and functional attributes [56], and our experiments did not distinguish between them. However, the fact that (a) *de novo* fatty-acid synthesis is elevated upon germline loss, and (b) *fasn-1;glp-1* exhibited dramatic lifespan suppression, suggest that lipogenic pathways perform crucial functions upon germline removal. While DGATs can help immure fats (normally designated for oocytes in fertile animals) into lipid droplets, what purpose is served by elevating fat production *per se*? The answer to this question is unknown, but based on emerging evidences, from vertebrate literature and worm studies, we postulate that enhanced *de novo* fatty-acid synthesis facilitates the production of lipophilic signals and ligands for transcription factors that enable the adaptation to germline loss. Fatty acids have been known to serve as signaling molecules for long. But, recent reports have begun to emphasize the importance of the ‘source’ of lipid signals. For instance, mice incapable of synthesizing ‘new fat’ due to fatty-acid synthase (FAS/FASN-1) deletion in the liver or hypothalamus cannot activate PPAR*α*, with which NHR-49 shares functions [57, 58]. Multiple lines of evidence indicate that inter-tissue communication in worms, including *glp-1* mutants, involves activation of transcription factors by steroid ligands. For instance, DA activates DAF-12 [31] and UFAs activate SKN-1 in *glp-1* mutants [15]. Oleoyl-ethanolamide (OEA) binds NHR-80 and is essential for expression of NHR-80 and NHR-49 targets in fertile adults [59]. So, it is plausible that DAF-16-mediated elevation of *de novo* lipid synthesis helps produce ligands for factors that help adapt to germline depletion. Further studies will be needed to test this hypothesis.

We noticed that inactivation of individual lipogenic genes did not have major effects on steady state fat levels, even in cases where *glp-1* mutants’ lifespan was substantially reduced. For instance, *fasn-1(fr7)* that abrogated *glp-1* longevity completely, and *pod-2* RNAi that significantly shortened lifespan, had considerably smaller effects on ORO levels in young *glp-1* adults, and the depletion was not retained in older animals (Fig 5 and S6 Fig). Both *fasn-1(fr8)* and *fasn-1* RNAi had strong lifespan effects, but only the mutation caused significant reduction in ORO labeling (Fig 5A and S7A–S7C Fig). One possible explanation for this perplexing observation is genetic redundancy, at least amongst the DGATs. Alternatively, these observations align with a growing body of research suggesting that steady-state fat levels and lifespan are not directly correlated [27, 29, 30]. Indeed, it is illustrated by the fact that germline-less animals and IIS mutants, both have higher fat but are healthier and longer-lived than their leaner, fertile counterparts [32, 60]. Interventions that increase fly lifespan, such as reduced IIS and TOR inhibition, also elevate fat [61, 62]. Similarly, ‘metabolically healthy obese’ individuals are noteworthy because they retain excessive weight without developing metabolic disorders [63]. Together with these observations, our data emphasize that the relationship between adiposity and lifespan is nuanced and multi-layered.

### The coupling of lipid synthesis and degradation in germline-less mutants

Besides demonstrating that lipogenic processes are augmented upon germline loss, our study also identified multiple lipolytic genes, including putative lipases. It has been reported by others that lipase activity is increased in germline-less adults dependent upon DAF-16, and our previous study suggested that β-oxidation is similarly augmented. Taken together, these observations imply that lipogenesis and lipolysis are concurrently amplified following germline depletion, although it remains to be demonstrated directly. Lipid turnover has been observed in other organisms under conditions of metabolic flux. Flies and mice undergoing dietary restriction (DR) display enhanced fatty acids turnover [64, 65]. Pertinently, inhibition of acetyl CoA carboxylase (dACC), the fly POD-2 homolog, abrogates DR-mediated longevity [65]. It is plausible that a similar large-scale turnover of cellular lipids following loss of the germline helps worms preserve metabolic homeostasis. Since our experiments did not gauge lipid turnover directly, further studies are needed to test this possibility. These processes may also be important for maintaining metabolic homeostasis in normal animals, since RNAi inactivation of some of the lipid genes shortened wild-type lifespans as well (S10 Table). Importantly, our study has identified multiple lipid-metabolic genes and pathways involved in the adaptive response to germline loss, many of which are orthologous to human genes, including ones implicated in diseases (S11 Table).

### The negative transcriptome: Genes repressed by DAF-16 and TCER-1

Active protein synthesis is critical for successful reproduction—interventions that impair translation often result in sterility. Similarly, a host of somatic genes and proteins are essential for successful procreation. If reproduction is prevented, an inability to reduce protein synthesis or stop the somatic programs that support reproduction can cause metabolic disarray. Our data implicate DAF-16 and TCER-1 as the enforcers of these repressive functions in *glp-1* mutants. The enrichment of ribosomal genes in the DAF-16-Specific DOWN class implies that the increased longevity of germline-less animals may be attributable, in part, to a global decrease in translation rates, a phenomenon associated with increased lifespan in many organisms, including worms [66, 67]. Accordingly, reduced protein synthesis in dietary-restricted worms and, in a DAF-16-dependent manner, in *daf-2* mutants has been observed [68]. Furthermore, the kinase TOR, that promotes ribosome biogenesis and translation, is down regulated in *glp-1* mutants by DAF-16 [13]. It remains to be investigated if DAF-16 represses ribosomal gene expression directly, or through TOR inhibition. TCER-1, on the other hand, represses the somatic gene-expression program of reproduction in *glp-1* mutants, and in addition, appears to impede the expression of many anti-longevity genes. Expectedly, there was some overlap between the two classes. Of the 13 TCER-1-Specific Class B genes whose inhibition increased wild-type lifespan, two are associated with fertility defects (*hel-1 and pcf-11*). Similarly, four of the 37 genes with sterility defects have been reported to be long-lived (Table 1). Unlike DAF-16, TCER-1 represses the expression of more genes than it upregulates (366 *vs*. 213) so its predominant transcriptional function may be that of a repressor, similar to TCERG1 [26]. The two factors also appear to exert opposite effects on a significant number of UP genes, including *lipl-1* and *lipl-2*. While RNA-Seq identified both genes as DAF-16-Specific UP and TCER-1-Specific DOWN targets, Q-PCRs showed that *lipl-2* is repressed by TCER-1 and *lipl-1* is upregulated. Also surprisingly, both genes promoted *glp-1* mutants’ longevity. Presently, the reason for this paradox, and the relevant importance of *lipl-1 vs*. *lipl-2*, is unclear. Further studies are needed to unravel the mechanism by which TCER-1 and DAF-16 mediate transcriptional activation *vs*. repression, and to understand their mutual interactions in influencing the expression of the ‘Opposite’ targets.

### Role of TCER-1 in balancing reproduction and longevity

*tcer-1* mutants were impaired in all aspects of reproduction including the number of progeny produced and the fraction that developed successfully. They manifested germ-cell defects, the most striking of which was germline heterochrony, and deficits in sperm and oocytes, whereas, a previous study that assayed only sterility reported no major defects [69]. *tcer-1* is expressed in both the soma and germline, and, since we did not perform tissue-specific knockdowns, the somatic contribution of TCER-1 to germline defects cannot be ruled out. Nonetheless, our experiments clearly demonstrate its necessity for optimal fertility. Thus, TCER-1 appears to perform two antithetical functions- in normal, fertile animals, it facilitates reproductive success, possibly by mediating germ-cell proliferation and ensuring gamete maturation in coordination with the somatic development. But, when the germline is eliminated, the somatic TCER-1 protein represses the expression of (somatic) genes that support reproduction. In addition, and together with DAF-16, it triggers the increased expression of lipid catabolic and anabolic genes that likely allows the animal to retain metabolic equilibrium and leads to increased lifespan. This discovery of TCER-1’s molecular adaptability, in promoting or repressing fertility based on the physiological requirement of the animal, provides a unique mechanistic insight into the cross talk between procreation and length of life (Fig 7I–7K). Notably, TCERG1 is expressed in mice and human oocytes [53, 70] and its levels are reduced in oocytes of menopausal women [53]. It will be interesting to investigate if TCER-1’s role in balancing fertility and longevity is conserved across evolutionary time scales.

## Methods

### Worm strains and culture

Animals were grown and maintained at 20°C using standard techniques [71]. The strains used in this study are N2, AGP162 {*acs-22(hj26)I* outcrossed to Ghazi lab N2}, AGP163 {*acs-22(hj26)I; glp-1(e2141ts)III*}, AGP164 {outcrossed *dgat-2(hj44)V*}, AGP165 {*glp-1(e2141ts)III; dgat-2(hj44)V*}, AGP166 {outcrossed IG346 (*fasn-1(fr8)I; frIs7 (nlp29p*::*GFP+col-12p*::*dsRed)IV*}, AGP167 {*fasn-1(fr8)I; glp-1(e2141ts)III*}, CF512 {*fer-15(b26)II; fem-1(hc17)IV}*, CF1903 {*glp-1(e2141)III*}, CF2154 {*tcer-1(tm1452)II; glp-1(e2141)III}*, CF1880 *{daf-16(mu86)I; glp-1(e2141)III}*, CF2166 *{tcer-1(tm1452)II}* and CF1038 *{daf-16(mu86)I}*. Strains carrying mutations in TCER-1-Specific DOWN genes that were used in lifespan experiments are listed in S9 Table.

### RNA-sequencing and data analysis

RNA was isolated on the second day of adulthood, using the mirVana miRNA Isolation Kit (Ambion, AM1561), from approximately 5000 worms each of CF1903, CF2154 and CF1880 (two biological replicates) and was prepared for sequencing using the Illumina TruSeq RNA Sample Preparation Kit as per the manufacturer’s instructions. The samples were then multiplexed prior to cluster formation and subjected to 50 base pair single-end sequencing on a Hiseq 2500 Illumina sequencer (Tufts University Genomics Core). The data was analyzed using the bioinformatics tools available at the Galaxy project [34]. Using FASTQ Groomer, the raw sequencing reads were initially converted to the Sanger FASTQ format that is compatible with TopHat Junction Mapper (1.5.0), the splice-junction mapping tool. Tophat *(*single end mating, default parameters) was then used to align the RNA-seq reads onto the *C*.*elegans* reference genome (WS190/*ce6*). The *S*equence *A*lignment *M*ap (SAM) format Tophat-output files were used as input for assembling the transcripts using Cufflinks and Cuffmerge (0.0.5). Cufflinks was run using 300000 as the max intron length, without quartile normalization and correcting for bias. Cufflink normalized and quantified the data to produce *F*ragments *P*er *K*ilobase of exon per *M*illion fragments mapped (FPKM). To facilitate combinatorial pairwise sample comparison, RNA-seq data was pooled with the reference annotation file using the meta-assembler, Cuffmerge, to result in a singular merged *G*ene *T*ransfer *F*ormat (GTF) file of all the transcripts. The Cuffdiff Differential Gene Expression tool (version 0.0.5) was used to calculate differential gene expression using a false discovery rate of 0.05, a minimum alignment count of 100 and with bias correction to obtain gene and transcript expression level data along with fold change (in log_2_ scale) and *P* values (raw and corrected for multiple testing). In the final lists of differentially regulated genes, a few loci had multiple genes mapping to them that necessitated manual curation (highlighted in blue in S1 Table).

### DAVID and REVIGO analyses

The Wormbase identifiers of the gene lists with the significantly and differentially expressed genes obtained from RNA-Seq analysis were uploaded on the publically available bioinformatic platform, *D*atabase for *A*nnotation, *V*isualization and *I*ntegrated *D*iscovery (DAVID) to identify enriched gene groups [39, 40]. The Functional Annotation Chart tool was used to identify the most overrepresented Gene Ontology terms associated with a given gene list, and these categories were probed for enriched gene groups reported as Gene Ontology (GO-BP) groups. REVIGO analysis was performed on the DAVID GO-BP groups to obtain summarized GO-BP-IDs [41].

### Q-PCRs

Worm RNA was isolated as described above, DNAse treated (DNAse kit, Sigma #AMPD1) and reverse transcribed into cDNA (Protoscript m-MuLV First Strand cDNA Synthesis kit, NEB #E6300S) according to the manufacturer's instructions. Quantitative real-time PCRs were performed using an Applied Bio Systems 7300 Real Time PCR System employing Sybr Green chemistry (SensiMix SYBR Hi-ROX kit, Bioline #QT-605). Gene expression data were normalized to housekeeping gene *rpl-32 (*and in many instance relative to *pmp-3* and *Y45F10D*.*4* as well) after confirming that *rpl-32* was expressed at the same level in all the strains. All data reported here were obtained by combining results from at least three independent biological replicates, each comprising 2–4 technical repeats. All primer sequences are listed in S12 Table.

### Lifespan assays

All lifespan experiments were carried out at 20°C unless otherwise noted. CF512, and temperature sensitive strains carrying the *glp-1* mutation, were initially grown at 20°C for 2-4hrs, transferred to 25°C till day 1 of adulthood, then returned to 20°C for the remainder of life. For RNAi experiments, worms were grown on *E*. *coli OP50*-seeded plates till mid-to-late L4 stage and then transferred to plates seeded with *E*. *coli HT115* bacteria carrying an empty control vector (pAD12) or relevant RNAi clones.

### Survival analysis

Survival curves were generated based on the Kaplan-Meier method using STATA 10.0 and 8.0 (Stata Corporation), or the online tool, OASIS (http://sbi.postech.ac.kr/oasis). Statistical significance was calculated using the non-parametric log-rank Mantel-Cox method. The students’ t-test was used to calculate significance for the Q-PCR data and the Mann-Whitney test was used to calculate statistical power for the reproductive health assays. The statistical significance of the overlap between two gene sets was calculated using the hypergeometric probability formula with normal approximation available as a program at nemates.org (http://nemates.org/MA/progs/overlap_stats.html).

### Stable isotope labeling, lipid purification and estimation

Gravid adults were bleached to obtain approximately 15000 eggs per strain and transferred to NGM plates seeded with *E*. *coli* OP50, incubated at 20°C for 2-4hrs and then transferred to 25°C. Animals were incubated at this temperature and collected either at the L4 stage (for comparing N2 and *glp-1* strains) or kept at 25°C till day 1, moved to 20°C thereafter and collected on day 2 of adulthood. For labeling, the collected animals were transferred for 6 hours to stable isotope plates, harvested, washed in M9 three times and frozen in a dry ice/ethanol bath and stored at -80°C until processed. Total lipids were extracted and purified as previously described [45]. Purified lipids were dried under nitrogen, re-suspended in methanol/2.5% H_2_SO_4_ and incubated for 1 h at 80°C to create fatty acid methyl esters (FAMEs) that were analyzed by gas chromatography/mass spectrometry (GC/MS) (Agilent 5975GC, 6920MS). TAG levels were determined as the NL/PL ratio based on the total abundance of the NL and PL fatty acids and recovery of the following internal standards: tritridecanoin (Nu-Chek Prep) and 1,2-diundecanoyl-*sn*-glycero-3-phosphocholine (Avanti Polar Lipids). Similar results were obtained upon estimating the ratio of TAGs to total TAGs + PLs. The total abundance of NL and PL fractions and analyses of these data are shown in S8 Fig.

### Fatty acid composition and *de novo* fatty acid synthesis analysis

The relative abundance of fatty acids in each class was determined for all the major fatty acid species in the nematode as previously described [45]. To quantify TAG and Phospholipid (PL) yields, total PL and TAG abundance was corrected for losses using added standards and data presented as a TAG:PL ratio, determined by measuring the sum of all major fatty acids found in TAGs versus the sum of the major fatty acids found in PLs. *de novo* fatty acid synthesis was calculated through a series of described equations which allow for the quantification of the amount of each fatty acid species generated from synthesis, distinguished by an isotope pattern from a mixture of ^12^C and ^13^C [45]. Numbers reported here represent the amount of ^13^C-labeled fatty acids derived from synthesis when compared to the total amount of fatty acids newly incorporated into the animal (Synthesized FA + Absorbed FA).

### ORO staining

ORO staining was done as described earlier [19, 32] using 30–40 animals per strain for each of two or three trials. Animals were mounted and imaged with using a Leica M165FC microscope equipped with a Retiga 2000R camera (Q Imaging). Images were captured with the QCapture Pro7 software (Q Imaging) and quantified using ImageJ software (NIH).

### Statistical analyses

To compare lipid levels between multiple genetic contexts, ORO staining results were converted into the fraction *F* of the area of each worm that was labeled with ORO. Comparisons of *F* between different genetic contexts (wildtype, RNAi, knockout) were performed in the framework of generalized linear models, which accounts for 0 < *F* < 1, summarizes over the two biological replicates, and provides tests akin to the (paired) t-test. Specifically, we used the *glm* function in the R language (R Core Team 2015) with a quasibinomial family, thereby accounting for overdispersion (high variance) in the observed data. Hypotheses about differences in *F* were tested using the *multcomp* package [72], and results are reported as univariate p values (shown in the tables associated with S6 and S7 Figs).

### Reproductive health assays

Reproductive health was assessed using previously described assays [54] as well as new measures (oocyte ratio). All experiments were conducted at 20°C and when matricide/bagging occurred the animal was censored from the experiment on that day. The data presented is obtained from aggregation of three independent trials, in each of which at least 10–15 animals per strain were examined. Individual synchronized L4 hermaphrodites were moved to fresh plates on a daily basis till the end of the reproductive phase (reproduction cessation for a minimum of 2 days), and number of eggs produced each day counted to calculate fecundity (total number of eggs laid by a hermaphrodite during its reproductive phase). Each day, once the parent was moved to a fresh plate, the older plate with eggs was stored at 20°C overnight, and the number of hatched worms counted the following day to calculate brood size. The above two parameters were used to determine viability (ratio of the total number of eggs laid by a hermaphrodite in its lifetime to total number of eggs that hatched). Similarly, the number of oocytes laid each day was counted to obtain oocyte number. These data were used to estimate the oocyte ratio (ratio of total number of oocytes laid by an animal to the total number of viable eggs it produced). Oocyte production span is the distribution of the oocyte ratio on a daily basis for the length of time that an animal lays any brood (eggs and oocytes combined). This parameter was used to assess premature oocyte production.

### Reciprocal crosses to identify sperm and oocyte defects

N2 males were crossed with *tcer-1* hermaphrodites (oocytes lacking *tcer-1)* and *tcer-1* males (sperm lacking *tcer-1)* were crossed with N2 hermaphrodites. As controls, N2 males and hermaphrodites were crossed to each other. In each case, approximately 15 individual crosses were performed with a male: hermaphrodite ratio of 3:1. Only plates with successful crosses (~50% male progeny) were considered for subsequent analyses. Hermaphrodites and males were transferred to fresh plates every day, and the various measures of reproductive health described above were calculated from the plates on which eggs were laid.

### DAPI staining

Strains were maintained at 20°C, or shifted to 25°C as L4 larvae and grown for one or two generations as required, prior to fixation. All animals were hand picked off plates, washed in M9 prior to fixation with Carnoy’s fixative (60% ethanol, 30% chloroform and 10% glacial acetic acid). Whole animals were then stained with DAPI for 15 minutes, washed with PBST (0.1% Triton) and mounted in Prolong Gold with DAPI (Life Technologies, Inc.) prior to imaging on a Nikon A1 confocal with 0.2micron Z-section. Images were visualized using Velocity software (PerkinElmer).

## Supporting Information

S1 Fig Expression of *fasn-1* and *atgl-1* in germline-ablated animals. mRNA levels of *fasn-1* (A) and *atgl-1* (B) compared between wild-type (N2, gray), *glp-1* (green), *daf-16;glp-1* (red) and *tcer-1;glp-1* (orange) day 2 adults by Q-PCR. Error bars denote the standard error of the mean. No statistical significance was observed in unpaired, two-tailed t-tests.
(TIF)

S2 Fig DAF-16 mediates increase in MUFA levels following germline loss. **A-C.** Effect of *daf-16* and *tcer-1* mutation on fatty-acid desaturase and elongase genes in *glp-1* mutants. mRNA levels of *fat-5* (A), *elo-1* (B) and *elo-2* (C) were compared between wild-type (N2, gray), *glp-1* (green), *daf-16;glp-1* (red) and *tcer-1;glp-1* (orange) day 2 adults by Q-PCR. *fat-6* upregulation, and *fat-7* downregulation, in *glp-1* mutants has previously been shown to be partially *daf-16*-dependent [18, 19]. **D-G.** Germline-less animals exhibited increased levels of UFAs. Fatty-acid compositions of neutral (D, F) and phospholipid (E,G) fractions compared through gas chromatography/mass spectrometry (GC/MS) between N2 (gray) and *glp-1* (green) late L4/early day 1 adults. *glp-1* mutants exhibit a significant increase in MUFA levels in the neutral lipid pool (NL) and PUFAs in phospholipids (PL), as compared to wild type. Cyclopropane fatty acids (CFAs) are decreased in both fractions, whereas, monomethyl branched-chain fatty acids (mmBCFA) levels are increased in the NL fraction of *glp-1* mutants but decreased in PL. The level of fatty acids with 18 carbon chain or longer (≥18C) is increased in both lipid fractions of *glp-1* mutants in keeping with the upregulation of *‘elo’* genes’ expression, and the level of fatty acids with less than 18 carbon chains (<18C) is correspondingly decreased. Data for individual fatty acids are shown in F, G. **H-K.** *daf-16* mutation reduced MUFA levels in neutral lipids. Neutral (H, J) and phospholipid (I, K) fatty acids compared by GC/MS between *glp-1* (green), *daf-16;glp-1*(red) and *tcer-1;glp-1*(orange) day 2 adults. *daf-16;glp-1* mutants show reduced MUFAs and elevated CFAs in both lipid fractions. There was no statistically significant difference between the three strains with respect to the carbon chain lengths of fatty acids. Changes observed in individual fatty acids are shown in J,K. Error bars denote the standard error of the mean. Asterisks represent the statistical significance of differences observed in an unpaired, two-tailed t-test with P values 0.05 (*), 0.005 (**) or < 0.0005 (***). Green asterisks indicate the comparison between N2 and *glp-1* whereas, red and orange asterisks depict the comparisons between *glp-1* and *daf-16;glp-1* or *tcer-1;glp-1*, respectively.
(TIF)

S3 Fig DAF-16 represses the expression of genes involved in protein translation following germline loss. **A.** List of genes included in the Gene Functional group with second highest enrichment score obtained through DAVID analysis of DAF-16-Specific DOWN genes. Genes encoding large ribosomal subunits, small ribosomal subunits and other translation factors are highlighted in light orange, pink and olive respectively. **B, C.** Graphic representation of the gene-function categories obtained through DAVID (B) and REVIGO analyses (C) of DAF-16-Specific DOWN genes. Translation is the largest functional category enriched. Additional details are shown in S4F, S4G and S5G Tables.
(TIF)

S4 Fig *tcer-1* mutants exhibit reduced fertility and germline defects. **A.** Oocyte span is increased in *tcer-1* mutants (orange) grown at 20°C as compared to wild type worms (N2, gray) and *daf-16* (red) mutants. **B.** Oocyte span in N2 males crossed to *tcer-1* mutant hermaphrodites (vertical striped bars) and *vice versa* (diagonal striped bars), compared to N2 males and hermaphrodites crossed to each other (gray). **C:** Quantification of germline defects seen in *tcer-1* mutants. The apparent rescue of the switch defect in *tcer-1* animals from day 1 to day 4 at 20°C reveals that the animals are delayed in the switch, but ultimately manage to accomplish oogenesis. In contrast, the lack of rescue in older animals at 25°C reflects a more severe defect in gonad morphogenesis. The number of gonad arms tested for each strain and condition (n) is shown above the respective bars. **D-E.** Images of DAPI-stained day 1 N2 (D) worms and *tcer-1* mutants (E) illustrate the reduced germline size in the latter. No difference was observed in the overall size of the animals.
(TIF)

S5 Fig Expression of transcription factors identified as UP genes DAF-16/TCER-1 dataset in germline-ablated animals. mRNA levels of *hlh-30*, *daf-12*, *skn-1*, *atfs-1* and *mdt-15* compared between wild-type (N2, gray), *glp-1* (green), *daf-16;glp-1* (red) and *tcer-1;glp-1* (orange) day 2 adults by Q-PCR. Error bars denote the standard error of the mean. Asterisks represent the statistical significance of differences observed in an unpaired, two-tailed t-test with P values 0.05 (*) or < 0.0005 (***). Green asterisks indicate the comparison between N2 and *glp-1* whereas, red ones depict the comparisons between *glp-1* and *daf-16;glp-1*.
(TIF)

S6 Fig Effect of *dgat-2(hj44)*, *acs-22(hj26) and fasn-1(fr8)* mutations on fat levels of *glp-1* mutants. **A, B.** Quantification of lipid levels compared between different strains through ORO staining on days 4 (A) and day 8 (B) adults grown on *E*. *coli OP50*. The box plots illustrate data from two biological replicates tested for each strain. The tables summarize the P values for the comparisons between ORO levels shown in A, B and Fig 5E (top) as well as the comparisons depicted in Fig 5F (bottom). See Methods for details of the statistical analyses.
(TIF)

S7 Fig Effect of RNAi inactivation of lipogenic genes on fat levels of *glp-1* mutants. Quantification of lipid levels compared between different strains through ORO staining on days 2 (A, D), 4 (B, E) and 8 (C, F) of adulthood of *glp-1* mutants grown on control empty vector (pAD12), *pod-2*, *mlcd-1* and *fasn-1* (A-C) and the ‘*dgat’* genes (C-E). Box plots depict data from the two biological replicates tested for each strain. The table summarizes the P values for all the comparisons undertaken for this dataset. See Methods‘ section for details of the statistical analyses.
(TIF)

S8 Fig Quantification of TAG and PL levels in mutant strains from GC-MS data. The total abundance of neutral lipid (NL) fatty acids and phospholipid (PL) fatty acids (A, B) in *glp-1* (green), *daf-16;glp-1* (red) and *tcer-1;glp-1* (orange) day 2 mutants was adjusted based on the recovery of internal lipid standards (tritridecanoin for NL and 1,2-diundecanoyl-*sn-*glycero-3-phosphocholine for PL) (C, D). The resulting data are shown in E and F and the graphical representation is in G and H, respectively. Because of the large numbers of worms needed to estimate fat content by GC-MS, the total amounts of NLs need to be normalized to compare between strains. As NL is largely made up by triacylglycerols (TAGs), we refer to this population from here on as TAG for simplicity. We normalized TAG levels with respect to PL abundance to obtain the data shown in Fig 4K, since PL levels do not change significantly between the strains and are believed to be a more robust standard to compare TAG levels across samples [73]. TAG/PL ratios most closely match the results obtained by fixative-based staining techniques too [19, 32, 60, 74]. Similar results were obtained when TAG levels were normalized relative to total PL + TAG (I and J) as an approximation of total lipid levels since TAGs and PLs are by far the largest contributors to the lipid population. Experiments were conducted on three biological replicates (BR). P values were determined by unpaired t-tests. In J, the asterisk represents the statistical significance of differences observed in an unpaired, two-tailed t-test with P values 0.05 (*). No significant differences were observed between the strains in the other comparisons shown in G, H and J.
(TIF)

S1 Table Lists of DAF-16 and TCER-1-regulated genes identified through RNA-Seq analysis. (XLSX)

S2 Table Comparison of overlap between DAF-16 downstream genes identified in this study with previously-identified DAF-16 targets. (PDF)

S3 Table Functional validation of RNA-Seq data through RNAi inactivation of UP genes in *glp-1* mutants. (XLSX)

S4 Table DAVID analysis of DAF-16 and TCER-1 targets identified in this study. (XLSX)

S5 Table REVIGO analysis of DAF-16 and TCER-1 targets identified in this study. (XLSX)

S6 Table Effect of *fasn-1(fr8)*, *dgat-2(hj44)* and *acs-22(hj26)* mutations on the longevity of *glp-1* mutants. (PDF)

S7 Table Effect of RNAi inactivation of lipid-metabolic genes on the longevity of *glp-1* mutants. (PDF)

S8 Table Effect of RNAi inactivation of TCER-1-Specific DOWN genes on the lifespan of wild-type surrogate strain *fer-15;fem-1*. (PDF)

S9 Table Effect of mutations in TCER-1-Specific DOWN genes on lifespan. (PDF)

S10 Table Effect of RNAi inactivation of lipid-metabolic genes on the longevity of wild-type worms. (PDF)

S11 Table Human orthologs of, and diseases associated with, the lipid-metabolic genes identified in this study. (PDF)

S12 Table Primers used in Q-PCR assays in this study. (PDF)

S1 Text List of supplementary references associated with S11 Table. (PDF)
