# Correction: DAF-16 and TCER-1 Facilitate Adaptation to Germline Loss by Restoring Lipid Homeostasis and Repressing Reproductive Physiology in *C*. *elegans*

**DOI:** 10.1371/journal.pgen.1006381

**Published:** 2016-10-07

**Authors:** Francis Raj Gandhi Amrit, Elizabeth Marie Steenkiste, Ramesh Ratnappan, Shaw-Wen Chen, T. Brooke McClendon, Dennis Kostka, Judith Yanowitz, Carissa Perez Olsen, Arjumand Ghazi

The Data Availability Statement for this article is incomplete. The statement should read: Raw data files are available from the SRA database, http://www.ncbi.nlm.nih.gov/sra, under accession numbers: SRX1754015, SRX1753861, SRX1753858, SRX1753857, SRX1753856 and SRX1753855. All other relevant data are within the article and its Supporting Information files.

## References

[1] Amrit FRG, Steenkiste EM, Ratnappan R, Chen S-W, McClendon TB, Kostka D, et al (2016) DAF-16 and TCER-1 Facilitate Adaptation to Germline Loss by Restoring Lipid Homeostasis and Repressing Reproductive Physiology in *C*. *elegans*. PLoS Genet 12(2): e1005788 doi: 10.1371/journal.pgen.1005788 26862916 10.1371/journal.pgen.1005788 PMC4749232

