# Supplementary figures and images for: DAF-16 and TCER-1 Facilitate Adaptation to Germline Loss by Restoring Lipid Homeostasis and Repressing Reproductive Physiology in C. elegans
Source: PLoS Genet. 2016 Feb 10;12(2):e1005788. doi: 10.1371/journal.pgen.1005788 (PMC4749232; doi:10.1371/journal.pgen.1005788)

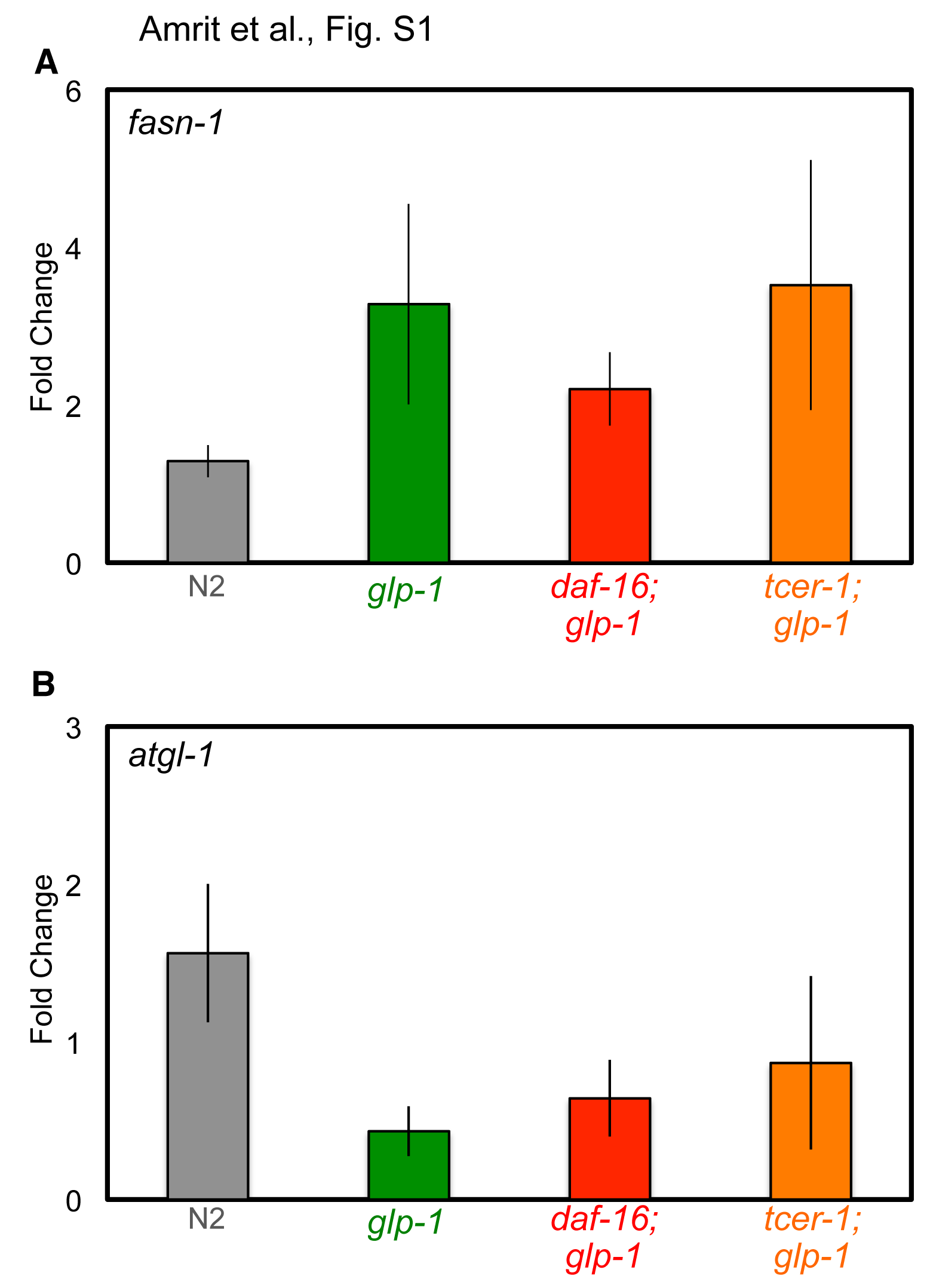

Supplement: S1 Fig — mRNA levels of fasn-1 (A) and atgl-1 (B) compared between wild-type (N2, gray), glp-1 (green), daf-16;glp-1 (red) and tcer-1;glp-1 (orange) day 2 adults by Q-PCR. Error bars denote the standard error of the mean. No statistical significance was observed in unpaired, two-tailed t-tests. (TIF) [file pgen.1005788.s001.tif]

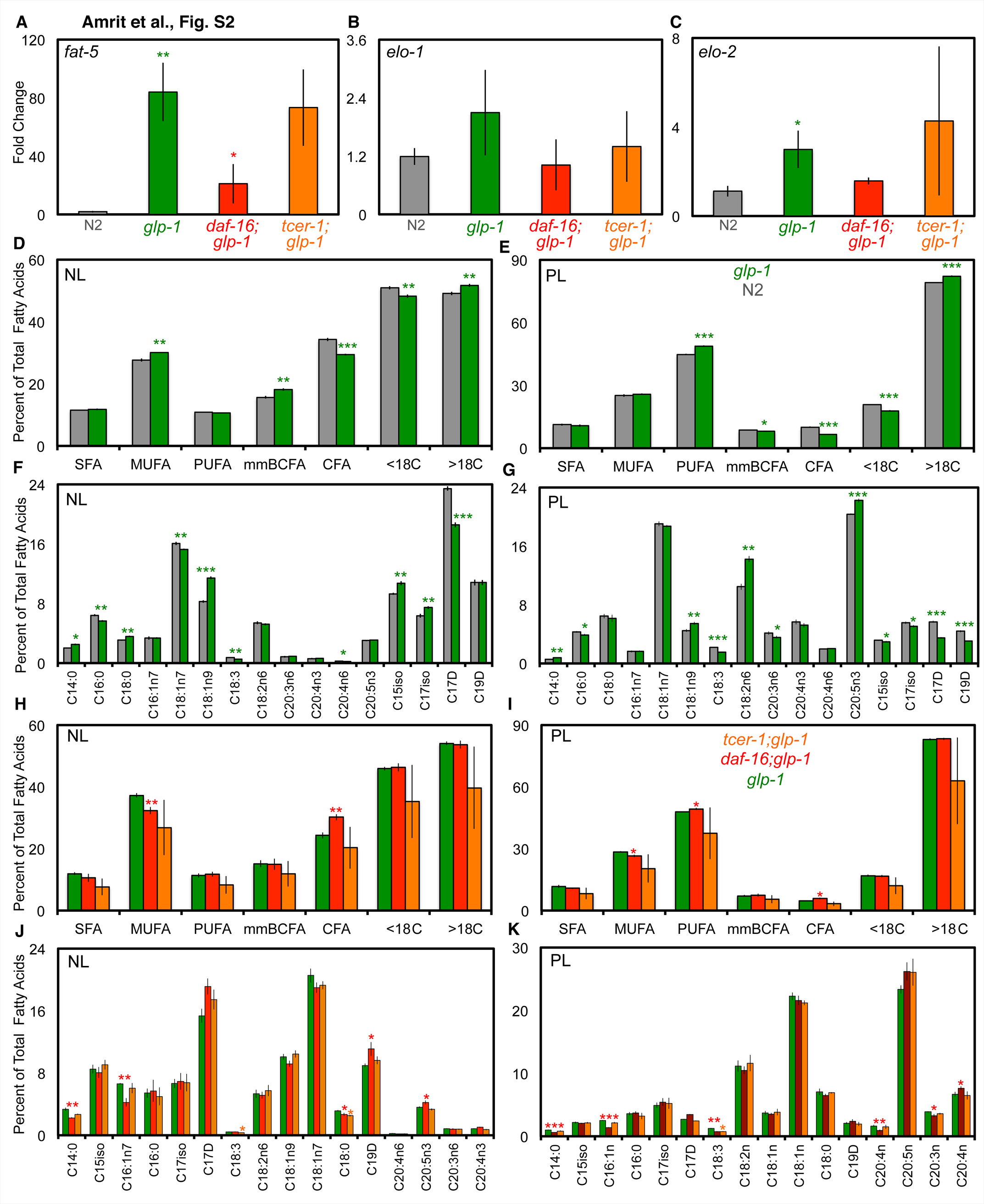

Supplement: S2 Fig — A-C. Effect of daf-16 and tcer-1 mutation on fatty-acid desaturase and elongase genes in glp-1 mutants. mRNA levels of fat-5 (A), elo-1 (B) and elo-2 (C) were compared between wild-type (N2, gray), glp-1 (green), daf-16;glp-1 (red) and tcer-1;glp-1 (orange) day 2 adults by Q-PCR. fat-6 upregulation, and fat-7 downregulation, in glp-1 mutants has previously been shown to be partially daf-16-dependent [18, 19]. D-G. Germline-less animals exhibited increased levels of UFAs. Fatty-acid compositions of neutral (D, F) and phospholipid (E,G) fractions compared through gas chromatography/mass spectrometry (GC/MS) between N2 (gray) and glp-1 (green) late L4/early day 1 adults. glp-1 mutants exhibit a significant increase in MUFA levels in the neutral lipid pool (NL) and PUFAs in phospholipids (PL), as compared to wild type. Cyclopropane fatty acids (CFAs) are decreased in both fractions, whereas, monomethyl branched-chain fatty acids (mmBCFA) levels are increased in the NL fraction of glp-1 mutants but decreased in PL. The level of fatty acids with 18 carbon chain or longer (≥18C) is increased in both lipid fractions of glp-1 mutants in keeping with the upregulation of ‘elo’ genes’ expression, and the level of fatty acids with less than 18 carbon chains (<18C) is correspondingly decreased. Data for individual fatty acids are shown in F, G. H-K. daf-16 mutation reduced MUFA levels in neutral lipids. Neutral (H, J) and phospholipid (I, K) fatty acids compared by GC/MS between glp-1 (green), daf-16;glp-1(red) and tcer-1;glp-1(orange) day 2 adults. daf-16;glp-1 mutants show reduced MUFAs and elevated CFAs in both lipid fractions. There was no statistically significant difference between the three strains with respect to the carbon chain lengths of fatty acids. Changes observed in individual fatty acids are shown in J,K. Error bars denote the standard error of the mean. Asterisks represent the statistical significance of differences observed in an unpaired, two-tailed t-test with [file pgen.1005788.s002.tif]

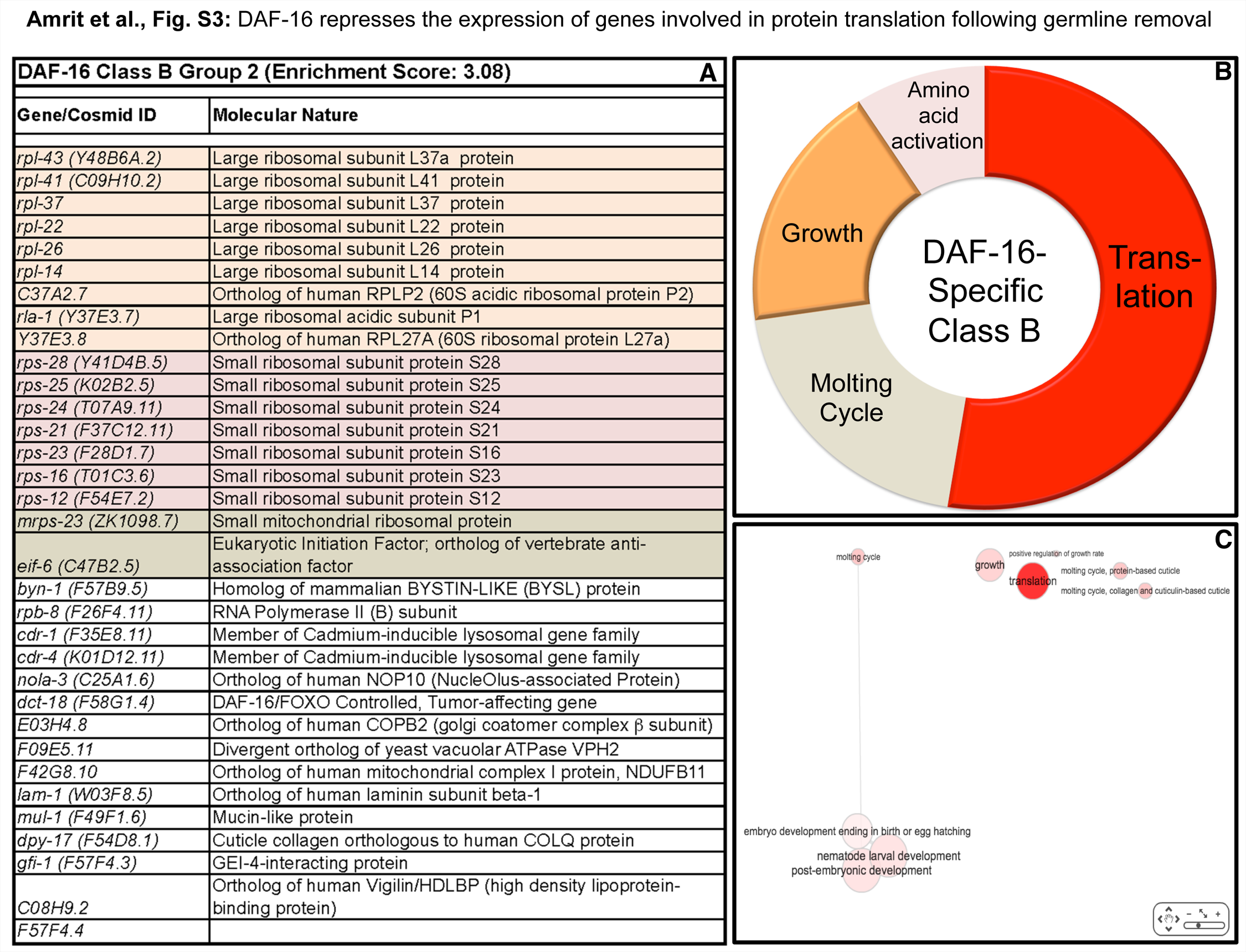

Supplement: S3 Fig — A. List of genes included in the Gene Functional group with second highest enrichment score obtained through DAVID analysis of DAF-16-Specific DOWN genes. Genes encoding large ribosomal subunits, small ribosomal subunits and other translation factors are highlighted in light orange, pink and olive respectively. B, C. Graphic representation of the gene-function categories obtained through DAVID (B) and REVIGO analyses (C) of DAF-16-Specific DOWN genes. Translation is the largest functional category enriched. Additional details are shown in S4F, S4G and S5G Tables. (TIF) [file pgen.1005788.s003.tif]

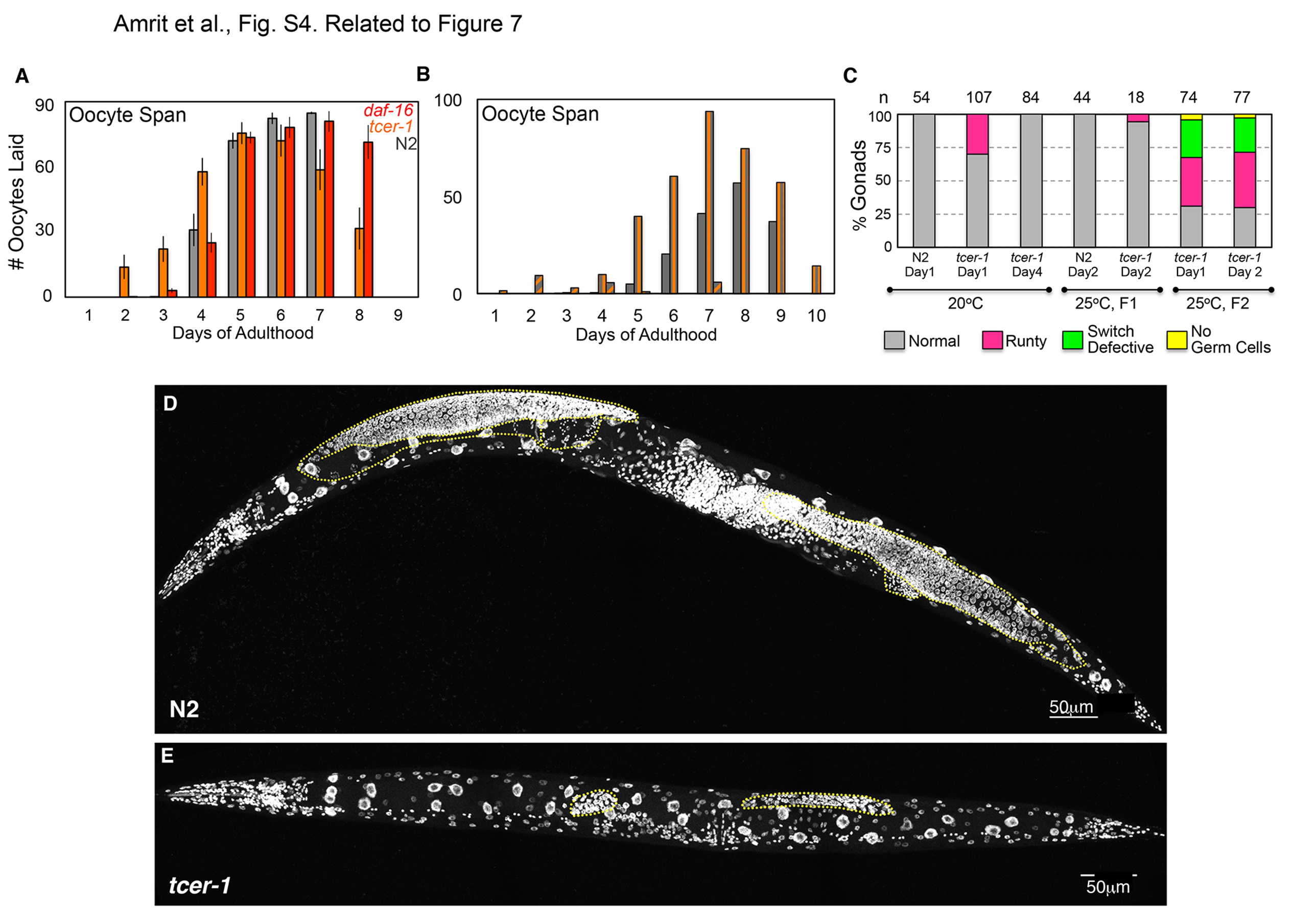

Supplement: S4 Fig — A. Oocyte span is increased in tcer-1 mutants (orange) grown at 20°C as compared to wild type worms (N2, gray) and daf-16 (red) mutants. B. Oocyte span in N2 males crossed to tcer-1 mutant hermaphrodites (vertical striped bars) and vice versa (diagonal striped bars), compared to N2 males and hermaphrodites crossed to each other (gray). C: Quantification of germline defects seen in tcer-1 mutants. The apparent rescue of the switch defect in tcer-1 animals from day 1 to day 4 at 20°C reveals that the animals are delayed in the switch, but ultimately manage to accomplish oogenesis. In contrast, the lack of rescue in older animals at 25°C reflects a more severe defect in gonad morphogenesis. The number of gonad arms tested for each strain and condition (n) is shown above the respective bars. D-E. Images of DAPI-stained day 1 N2 (D) worms and tcer-1 mutants (E) illustrate the reduced germline size in the latter. No difference was observed in the overall size of the animals. (TIF) [file pgen.1005788.s004.tif]

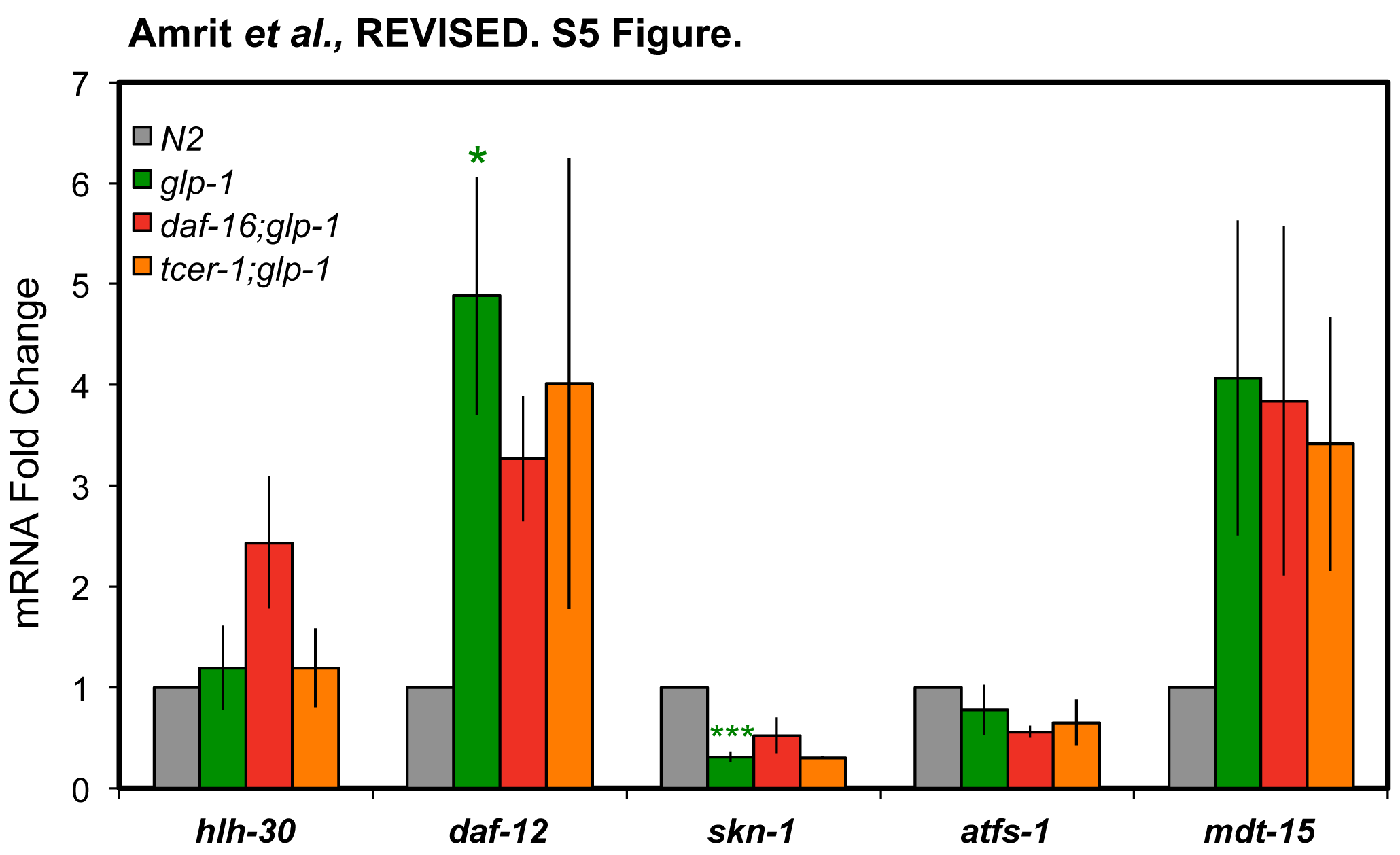

Supplement: S5 Fig — mRNA levels of hlh-30, daf-12, skn-1, atfs-1 and mdt-15 compared between wild-type (N2, gray), glp-1 (green), daf-16;glp-1 (red) and tcer-1;glp-1 (orange) day 2 adults by Q-PCR. Error bars denote the standard error of the mean. Asterisks represent the statistical significance of differences observed in an unpaired, two-tailed t-test with P values 0.05 (*) or < 0.0005 (***). Green asterisks indicate the comparison between N2 and glp-1 whereas, red ones depict the comparisons between glp-1 and daf-16;glp-1. (TIF) [file pgen.1005788.s005.tif]

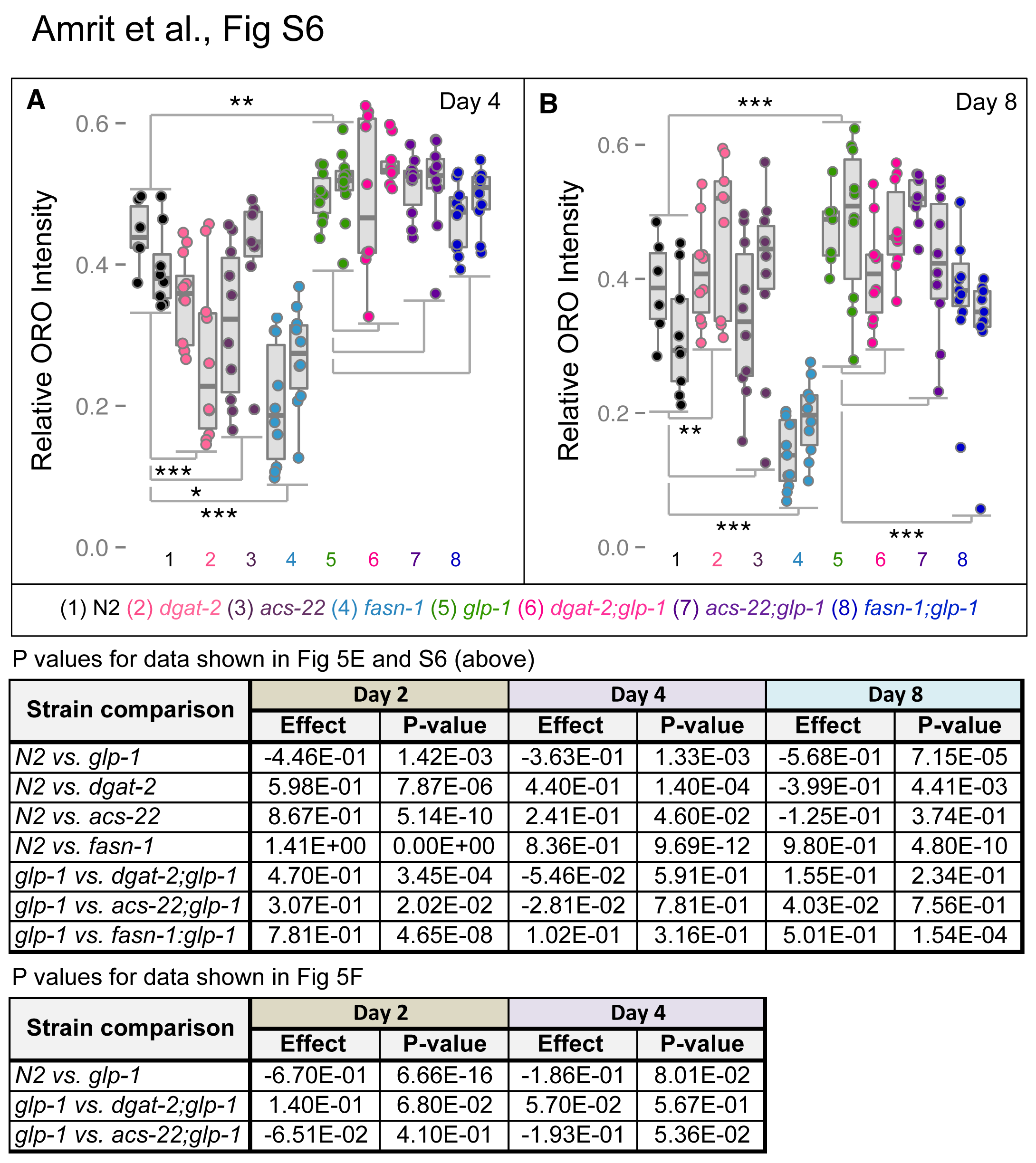

Supplement: S6 Fig — A, B. Quantification of lipid levels compared between different strains through ORO staining on days 4 (A) and day 8 (B) adults grown on E. coli OP50. The box plots illustrate data from two biological replicates tested for each strain. The tables summarize the P values for the comparisons between ORO levels shown in A, B and Fig 5E (top) as well as the comparisons depicted in Fig 5F (bottom). See Methods for details of the statistical analyses. (TIF) [file pgen.1005788.s006.tif]

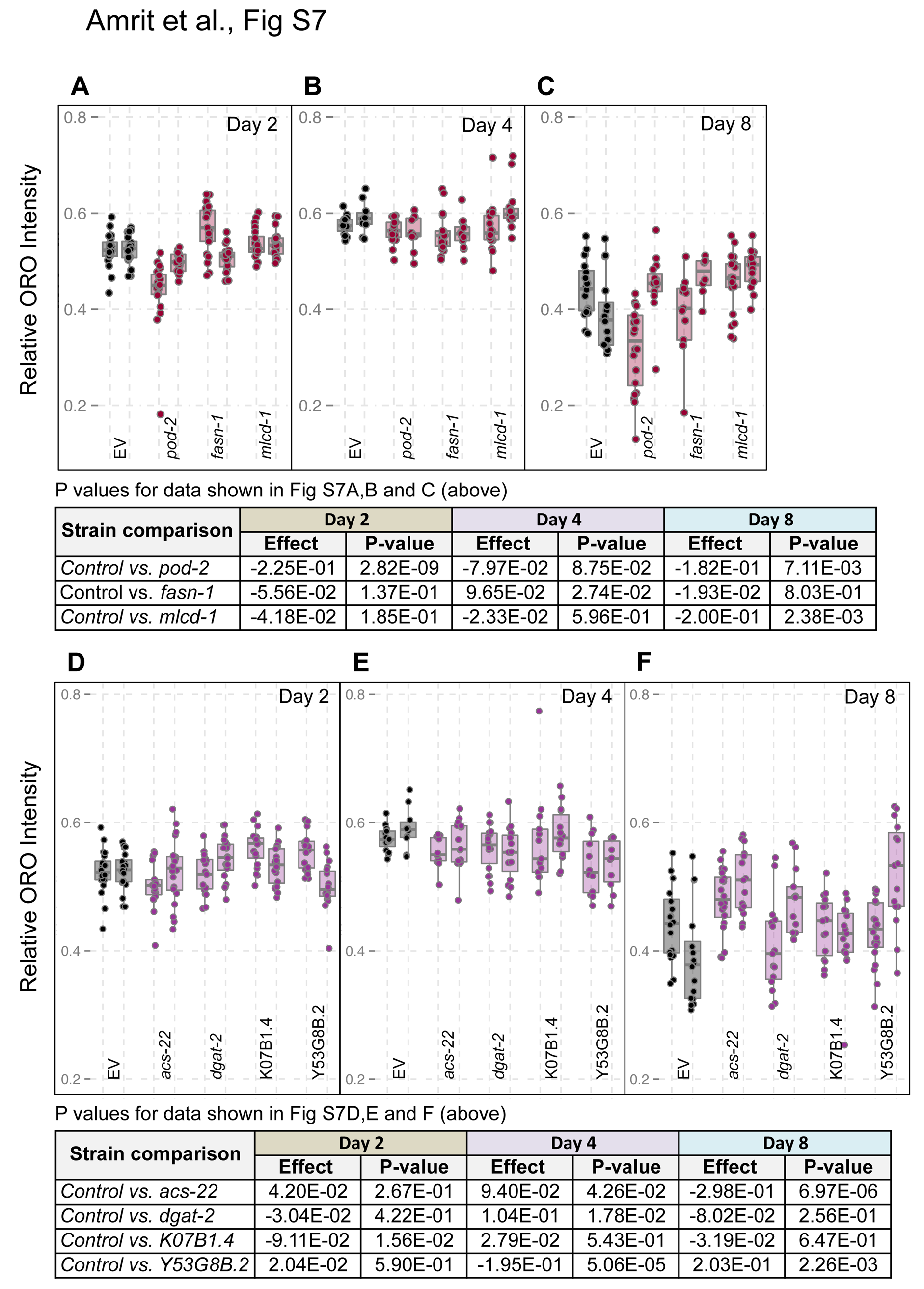

Supplement: S7 Fig — Quantification of lipid levels compared between different strains through ORO staining on days 2 (A, D), 4 (B, E) and 8 (C, F) of adulthood of glp-1 mutants grown on control empty vector (pAD12), pod-2, mlcd-1 and fasn-1 (A-C) and the ‘dgat’ genes (C-E). Box plots depict data from the two biological replicates tested for each strain. The table summarizes the P values for all the comparisons undertaken for this dataset. See Methods‘ section for details of the statistical analyses. (TIF) [file pgen.1005788.s007.tif]

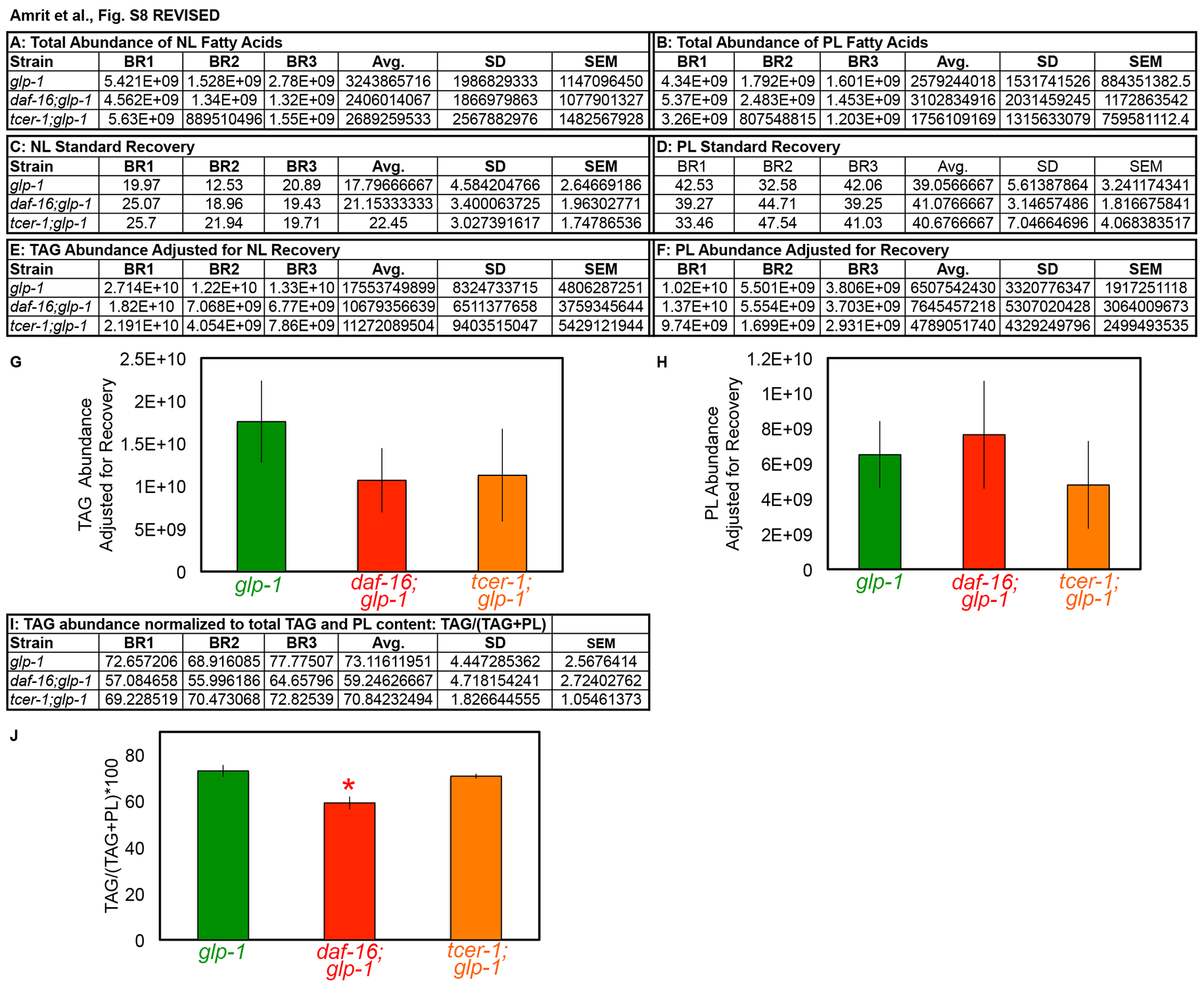

Supplement: S8 Fig — The total abundance of neutral lipid (NL) fatty acids and phospholipid (PL) fatty acids (A, B) in glp-1 (green), daf-16;glp-1 (red) and tcer-1;glp-1 (orange) day 2 mutants was adjusted based on the recovery of internal lipid standards (tritridecanoin for NL and 1,2-diundecanoyl-sn-glycero-3-phosphocholine for PL) (C, D). The resulting data are shown in E and F and the graphical representation is in G and H, respectively. Because of the large numbers of worms needed to estimate fat content by GC-MS, the total amounts of NLs need to be normalized to compare between strains. As NL is largely made up by triacylglycerols (TAGs), we refer to this population from here on as TAG for simplicity. We normalized TAG levels with respect to PL abundance to obtain the data shown in Fig 4K, since PL levels do not change significantly between the strains and are believed to be a more robust standard to compare TAG levels across samples [73]. TAG/PL ratios most closely match the results obtained by fixative-based staining techniques too [19, 32, 60, 74]. Similar results were obtained when TAG levels were normalized relative to total PL + TAG (I and J) as an approximation of total lipid levels since TAGs and PLs are by far the largest contributors to the lipid population. Experiments were conducted on three biological replicates (BR). P values were determined by unpaired t-tests. In J, the asterisk represents the statistical significance of differences observed in an unpaired, two-tailed t-test with P values 0.05 (*). No significant differences were observed between the strains in the other comparisons shown in G, H and J. (TIF) [file pgen.1005788.s008.tif]
