# Supplementary material for: DAF-16 and TCER-1 Facilitate Adaptation to Germline Loss by Restoring Lipid Homeostasis and Repressing Reproductive Physiology in C. elegans
Source: PLoS Genet. 2016 Feb 10;12(2):e1005788. doi: 10.1371/journal.pgen.1005788 (PMC4749232; doi:10.1371/journal.pgen.1005788)
Supplement: S2 Table — (PDF) [file pgen.1005788.s010.pdf]

Amrit *et al.,* Table S2: Comparison of DAF-16 and TCER-1 targets with previous DAF-16 genomic studies

1. Comparison with DAF-16 'Consensus' Target List (Tepper *et al., Cell*. 2013, 154(3):676-90. PMID: 23911329)

Consensus Data Classes	# Genes
DAF-16 +ve targets- Upregulated (correspond to UP class of this study)	1663
DAF-16 -ve target - Downregulated (correspond to DOWN class of this study)	1733

RNA-Seq Gene Class	"Consensus" Gene Class	Overlap	%	Statistical Significance	Representation Factor
DAF-16-Specific UP	DAF-16 +ve targets	162/349	46.42%	$p < 9.426e-80$	5.4
DAF-16-Specific DOWN	DAF-16 +ve targets	5/196	2.55%	$p < 4.646e-04$	0.3
Joint DAF-16 and TCER-1 UP	DAF-16 +ve targets	70/123	56.91%	$p < 1.946e-42$	6.6
Joint DAF-16 and TCER-1 DOWN	DAF-16 +ve targets	1/73	1.37%	$p < 0.011$	0.2
Opposite DAF-16 and TCER-1	DAF-16 +ve targets	44/60	73.33%	$p < 3.189e-34$	8.5
TCER-1-Specific UP	DAF-16 +ve targets	28/213	13.15%	$p < 0.016$	1.5
TCER-1-Specific DOWN	DAF-16 +ve targets	61/366	16.67%	$p < 4.296e-07$	1.9
DAF-16-Specific UP	DAF-16 -ve targets	12/349	3.44%	$p < 3.494e-05$	0.4
DAF-16-Specific DOWN	DAF-16 -ve targets	110/196	56.12%	$p < 1.991e-63$	6.2
Joint DAF-16 and TCER-1 UP	DAF-16 -ve targets	5/123	4.07%	$p < 0.030$	0.5
Joint DAF-16 and TCER-1 DOWN	DAF-16 -ve targets	41/73	56.16%	$p < 2.148e-24$	6.3
Opposite DAF-16 and TCER-1	DAF-16 -ve targets	21/60	35.00%	$p < 2.409e-08$	3.9
TCER-1-Specific UP	DAF-16 -ve targets	64/213	30.05%	$p < 1.215e-18$	3.3
TCER-1-Specific DOWN	DAF-16 -ve targets	49/366	13.39%	$p < 0.003$	1.5

2. Comparison with DAF-16 targets in *glp-1* mutants (McCormick *et al., Aging Cell*. 2012, 11(2):192-202. PMID: 22081913)

230 genes were identified as 'DAF-16-regulated' including both positive and negative targets

RNA-Seq Gene Class	McCormick Data	Overlap	% of McCormick *et al.*	Statistical Significance	Representation Factor
DAF-16 Targets	DAF-16 regulated genes	57/230	25%	**$p < 1.870e-35$**	**8**
Joint DAF-16 and TCER-1 Targets	DAF-16 regulated genes	28/230	12%	**$p < 4.820e-18$**	**8.4**
	Overall DAF-16 overlap	*85/230*	*37%*	***$p < 7.840e-55$***	***8.1***
TCER-1 Targets	DAF-16 regulated genes	10/230	4%	**$p < 0.227$**	**1.3**

3. Comparison with DAF-16 target list in IIS mutants (Murphy *et al., Nature*. 2003, 424(6946):277-83. PMID: 12845331)

Murphy *et al.,* Data Class	# Genes
DAF-16 +ve targets (Class 1) - Upregulated (correspond to Class A genes of this study)	259
DAF-16 -ve targets (Class 2) - Downregulated (correspond to Class B genes of this study)	250

RNA-Seq Gene Class	Murphy Data Class	Overlap	%	Statistical Significance	Representation Factor
DAF-16-Specific UP	DAF-16 +ve targets (Class 1)	53/349	15.19%	**$p < 1.692e-38$**	**10.3**
DAF-16-Specific DOWN	DAF-16 +ve targets (Class 1)	0/196			
Joint DAF-16 and TCER-1 UP	DAF-16 +ve targets (Class 1)	20/123	16.26%	**$p < 1.404e-15$**	**11.1**
Joint DAF-16 and TCER-1 DOWN	DAF-16 +ve targets (Class 1)	0/73			
Opposite DAF-16 and TCER-1	DAF-16 +ve targets (Class 1)	5/60	8.33%	**$p < 0.002$**	**5.7**
TCER-1-Specific UP	DAF-16 +ve targets (Class 1)	3/213	1.41%	**$p < 0.383$**	**1**
TCER-1-Specific DOWN	DAF-16 +ve targets (Class 1)	15/366	4.10%	**$p < 3.581e-04$**	**2.8**
DAF-16-Specific UP	DAF-16 -ve targets (Class 2)	1/349	0.29%	**$p < 0.040$**	**0.2**
DAF-16-Specific DOWN	DAF-16 -ve targets (Class 2)	15/196	7.65%	**$p < 1.372e-07$**	**5.4**
Joint DAF-16 and TCER-1 UP	DAF-16 -ve targets (Class 2)	0/123			
Joint DAF-16 and TCER-1 DOWN	DAF-16 -ve targets (Class 2)	14/73	19.18%	**$p < 1.647e-12$**	**13.5**
Opposite DAF-16 and TCER-1	DAF-16 -ve targets (Class 2)	5/60	8.33%	**$p < 0.002$**	**5.9**
TCER-1-Specific UP	DAF-16 -ve targets (Class 2)	14/213	6.57%	**$p < 2.245e-06$**	**4.6**
TCER-1-Specific DOWN	DAF-16 -ve targets (Class 2)	4/366	1.09%	**$p < 0.403$**	**0.8**

4. Comparison with DAF-16 'Isoform A and F' Target List (Chen *et al., Genetics*. 2015 201(2): 613-629. PMID: 26219299)

399 genes were identified as DAF-16A/F targets in the article. In actuality, the list included 450 genes because in some cases a genomic location identified in the RNA-Seq data included more than one gene. The comparisons below were performed with 450 genes.

RNA-Seq Gene Class	Chen et al., Gene Class	Overlap	%	Statistical Significance	Representation Factor
All DAF-16 targets	DAF-16 A/F targets	58/545	10.64%	$p < 1.619e-20$	4.2
All Joint DAF-16 and TCER-1 targets	DAF-16 A/F targets	62/256	24.22%	$p < 6.465e-43$	9.5
All TCER-1 targets	DAF-16 A/F targets	36/579	6.22%	$p < 8.793e-07$	2.4

5. Comparison with genes up-regulated in *glp-1* mutants (Steinbaugh *et al., eLife*. 2015; 4: e07836. PMID: 26196144)

Data Classes	# Genes
Genes upregulated in *glp-1* mutants	1306

RNA-Seq Gene Class	Gene Class	Overlap	%	Statistical Significance	Representation Factor
All DAF-16 targets	Genes activated by GSC removal	125/545	22.94%	$p < 2.808e-31$	3.1
All Joint DAF-16 and TCER-1 targets	Genes activated by GSC removal	85/256	33.20%	$p < 6.884e-34$	4.5
All TCER-1 targets	Genes activated by GSC removal	143/579	24.70%	$p < 1.278e-39$	3.3

Statistical significance and representation factor were calculated using http://nemates.org/MA/progs/overlap_stats.html. The total number of genes in the genome was set at 19682 for the first comparison (same as Tepper et al.,) and at 17611 (the number of good spots on the Kim lab full genome microarray) for the other two comparisons.
