# Supplementary material for: DAF-16 and TCER-1 Facilitate Adaptation to Germline Loss by Restoring Lipid Homeostasis and Repressing Reproductive Physiology in C. elegans
Source: PLoS Genet. 2016 Feb 10;12(2):e1005788. doi: 10.1371/journal.pgen.1005788 (PMC4749232; doi:10.1371/journal.pgen.1005788)
Supplement: S6 Table — (PDF) [file pgen.1005788.s014.pdf]

Amrit *et al.*, Table S6: Effect of *fasn-1*, *acs-22* and *dgat-2* mutations on lifespan of wild type worms a

Strain	Trial1						
	n= Obs/Tot	Mean ± SEM	% impact on *glp-1* lifespan	P *vs. glp-1*	P *vs. N2*	n= Obs/Tot	Mean ± SEM
N2 (Wildtype)	76/91	16.7 ± 0.7				39/84	20.0 ± 0.3
glp-1	76/85	26.5 ± 0.5			<0.0001	73/76	24.5 ± 0.9
fasn-1	62/75	19.1 ± 0.6		<0.0001	0.13	63/68	21.4 ± 0.3
fasn-1;glp-1	84/88	18.0 ± 0.8	-32	<0.0001	0.12	76/84	20.2 ± 0.8
dgat-2	75/88	22.6 ± 0.6		<0.0001	<0.0001	59/82	23.8 ± 0.2
dgat-2;glp-1	52/61	28.5 ± 1.0	+7	0.64	<0.0001	79/86	28.8 ± 0.3
N2 (Wildtype)	69/100	17.0 ± 0.7				57/74	19.3 ± 0.5
glp-1	92/96	21.0 ± 0.9			<0.0001	105/105	21.6 ± 0.9
acs-22	72/83	19.1 ± 0.5		<0.0006	0.1	83/102	18.8 ± 0.3
acs-22;glp-1	97/103	23.6 ± 0.6	+12	0.87	<0.0001	94/110	26.0 ± 0.2

...nd *glp-1* mutants

Trial 2		
% impact on *glp-1* lifespan	P *vs. N2*	P *vs. glp-1*
	0.04	
	0.19	0.004
-17.5	<0.0001	0.18
	0.0001	0.05
+17.5	<0.0001	0.12
	0.007	
	0.19	0.004
+20.3	<0.0001	0.18
