# Supplementary material for: DAF-16 and TCER-1 Facilitate Adaptation to Germline Loss by Restoring Lipid Homeostasis and Repressing Reproductive Physiology in C. elegans
Source: PLoS Genet. 2016 Feb 10;12(2):e1005788. doi: 10.1371/journal.pgen.1005788 (PMC4749232; doi:10.1371/journal.pgen.1005788)
Supplement: S7 Table — (PDF) [file pgen.1005788.s015.pdf]

Amrit *et al.*, Table S7: Effect of RNAi inactivation of lipid-metabolic genes on extended lifespan of *glp-1* mutants.

Gene Name (Cosmid)	RNA-Seq Class	Trial #1				Trial #2				Trial #3				Trial #4			
		n = obs/total	Mean +/- SEM	% Lifespan impact	P (*vs* pAD-12)	n = obs/total	Mean +/- SEM	% Lifespan impact	P (*vs* pAD-12)	n = obs/total	Mean +/- SEM	% Lifespan impact	P (*vs* pAD-12)	n = obs/total	Mean +/- SEM	% Lifespan impact	P (*vs* pAD-12)
pAD-12 (Empty control vector)		63/83	23.2+/-0.3			121/127	26.71+/-0.85			77/91	23.23+/-0.86			49/50	27.99+/-1.39		
daf-16 (R13H8.1)		79/79	15.2+/-0.3	-34.4827586	<0.0001	117/117	17.32+/-0.57	-35	<0.0001	83/110	15.77+/-0.36	-32	<0.0001	74/78	14.81+/-0.38	-47	<0.0001
tcer-1 (ZK1127.9)		98/103	18.3+/-0.9	-21.1206897	<0.0001	93/95	22.05+/-0.85	-17	<0.0001	75/85	20.08+/-0.77	-14	<0.0001	76/78	19.07+/-0.67	-32	<0.0001
pod-2 (W09B6.1)	DAF-16-Specific UP	86/94	17.3+/-0	-25.4310345	<0.0001	104/104	24.96+/-1.13	-7	0.812	83/91	22.34+/-0.90	-4	0.5163	64/65<br>^61/63	20.68+/-1.00<br>19.81+/-0.87	-26<br>-29	<0.0001<br><0.0001
mlcd-1 (F35G12.1)	DAF-16-Specific UP	42/43	15.8+/-0	-31.8965517	<0.0001	91/97	26.71+/-0.86	0	0.741	53/75	20.67+/-1.12	-11	0.0575				
fasn-1 (F32H2.5)	NR*	56/56	17.1+/-0.2	-26.2931034	<0.0001	140/140	20.88+/-0.69	-22	<0.0001	59/85	18.70+/-0.70	-20	0.0001	69/72	18.5+/-0.46	-34	<0.0001
dgat-2 (F59A1.10)	TCER-1-Specific UP	71/80	18.5+/-0.7	-20.2586207	<0.0001	58/61	20.41+/-1.18	-24	<0.0001	86/93	23.31+/-0.96	0	<0.0001	62/65	23.24+/-1.03	-17	0.0001
Y53G8B.2	Joint UP	59/71	20.7+/-0.5	-10.7758621	0.02	140/140	23.33+/-0.85	-13	0.0186	84/90	23.02+/-1.12	-1	0.6914	69/75	24.28+/-0.93	-13	0.0028
acs-22 (D1009.1)	DAF-16-Specific UP	123/123	19.7+/-1.3	-15.0862069	0.006	92/92	24.62+/-1.00	-8	0.1533	86/89	22.52+/-0.83	-3	0.2185				
K07B1.4	Joint UP	97/97	19.9+/-1	-14.2241379	0.0009	156/156	21.81+/-0.75	-18	<0.0001	72/83	22.61+/-1.05	-3	0.7701				
lipl-1 (F54F3.3)	Opposite	89/89	20.5+/-0.8	-11.637931	0.01	145/151	20.76+/-0.86	-22	0.0002								
lipl-2 (F46B6.8)	Opposite	63/74	18.9+/-0.6	-18.5344828	0.0001	122/127	22.69+/-0.95	-15	0.0351								
lipl-5 (ZK6.7)	Joint UP	78/79	20.2+/-0.5	-12.9310345	0.003	180/180	21.17+/-0.77	-21	0.0005					71/74	22.84+/-1.11	-18	0.0091
lips-10 (F14E5.5)	TCER-1-Specific UP	94/98	21+/-0.2	-9.48275862	0.01	89/90	21.72+/-0.98	-19	0.0001								
lips-14 (H17B01.3)	TCER-1-Specific UP	83/86	18.5+/-0.4	-20.2586207	<0.0001	81/81	22.25+/-1.11	-17	0.0040								
atgl-1 (C05D11.7)	DAF-16-Specific UP	75/82	15.7+/-0	-32.3275862	<0.0001	124/128	25.71+/-1.12	-4	0.1528	71/88	23.83+/-1.09	3	0.4526	58/60	21.52+/-1.01	-23	<0.0001

*Not represented ^ Gene tested twice in the same experiment
