# Supplementary material for: DAF-16 and TCER-1 Facilitate Adaptation to Germline Loss by Restoring Lipid Homeostasis and Repressing Reproductive Physiology in C. elegans
Source: PLoS Genet. 2016 Feb 10;12(2):e1005788. doi: 10.1371/journal.pgen.1005788 (PMC4749232; doi:10.1371/journal.pgen.1005788)
Supplement: S8 Table — (PDF) [file pgen.1005788.s016.pdf]

Amrit *et al.*, Table S8: Effect of RNAi inactivation of TCER-1-Specific DOWN genes on lifespan of wild-type surrogate strain *fer-15;fem-1*

Gene Name (Cosmid)	Trial #1				Trial #2				Trial #3			
	n = obs/total	Mean +/- SEM	% Lifespan Increase	P *(vs* pAD12)	n = obs/total	Mean +/- SEM	% Lifespan Increase	P *(vs* pAD12)	n = obs/total	Mean +/- SEM	% Lifespan Increase	P *(vs* pAD-12)
pAD12 (Empty control vector)	57/80	14.1 +/- 0.6			76/76	12.6 +/- 0.3			31/40	14.4 +/- 0.7		
daf-2 (Y55D5A.5)	73/76	22.1 +/- 0.9	56	<0.0001	80/84	16.4 +/- 0.6		<0.0001	56/60	19.8 +/- 1.0	38	0.01
F56D3.1	75/77	16.0 +/- 0.6	13	0.03	69/72	13.8 +/- 0.5	10	0.05				
gst-24 (F37B1.1)	70/70	15.9 +/- 0.5	13	0.03	60/60	14.18 +/- 0.5	12	0.01				
numr-1 (F08F8.5)	75/87	16.6 +/- 0.4	18	0.003	66/67	14.3 +/- 0.4	14	0.002				
col-117 (T28C6.4)	89/89	16.1 +/- 0.4	14	0.01	61/62	15.2 +/- 0.5	21	<0.0001				
col-3 (T28C6.6)	76/78	16.0 +/- 0.6	13	0.03	90/92	13.9 +/- 0.3	10	0.04				
dhs-14 (R05D8.8)	77/79	16.5 +/- 0.5	17	0.005	47/49	13.1 +/- 0.5	5	0.78				
ilys-3 (C45G7.3)	87/88	14.9 +/- 0.4	6	0.39	73/74	13.9 +/- 0.4	10	0.03	69/71	13.4 +/- 0.5	-7	0.05
spp-18 (F27C8.4)	71/74	16.0 +/- 0.6	13	0.03	53/54	13.2 +/- 0.4	5	1	49/50	14.5 +/- 0.8	1	0.69
lys-4 (F58B3.1)	78/83	17.2 +/- 0.5	22	0.0003	78/80	14.5 +/- 0.4	15	0.003	45/52	16.7 +/- 0.7	16	0.32
pAD12 (Empty control vector)	66/74	19.2 +/- 0.4										
daf-2 (Y55D5A.5)	71/75	27.1 +/- 0.6	41	<0.0001								
pcf-11 (R144.2)	39/43	12.1 +/- 0.3	-37	<0.0001	64/67	9.6 +/- 0.3	-23	<0.0001	69/69	7.1 +/- 0.1	-51	<0.0001
lys-10 (F17E9.11)	20.9	20.9 +/- 0.4	9	0.0008	79/84	13.7 +/- 0.4	9	0.03	69/70	15.0 +/- 0.5	4	0.45
ilys-2 (C45G7.2)	59/60	19.8 +/- 0.4	3	0.08	70/70	12.2 +/- 0.6	-3	0.75	62/64	12.7 +/- 0.6	-12	0.04
Y49G5A.1	46/75	21.1 +/- 0.4	10	0.002	59/59	12.3 +/- 0.6	-2	1	46/49	15.6 +/- 0.7	9	0.8
F41F3.3	75/75	19.9 +/- 0.4	4	0.18	61/61	10.8 +/- 0.5	-14	0.11	55/56	13.9 +/- 0.5	-3	0.2
Y41C4A.11	75/75	19.3 +/- 0.3	ne	0.43	46/46	13.2 +/- 0.6	5	0.13	60/60	13.3 +/- 0.7	-8	0.07
hel-1 (C26D10.2)	72/72	18.4 +/- 0.4	-4	0.3	77/81	12.1 +/- 0.5	-4	1	55/56	14.7 +/- 0.7	2	0.39
T27A1.2	56/75	19.3 +/- 0.3	ne	0.06	48/49	13.7 +/- 0.5	9	0.11				
F57H12.6	51/60	20.9 +/- 0.5	9	0.01	79/80	13.8 +/- 0.4	10	0.006				
col-81 (F38A3.10	60/60	21.8 +/- 0.4	13	<0.0001	72/73	14.5 +/- 0.4	15	0.0001				

ne: no effect
