# Supplementary material for: DAF-16 and TCER-1 Facilitate Adaptation to Germline Loss by Restoring Lipid Homeostasis and Repressing Reproductive Physiology in C. elegans
Source: PLoS Genet. 2016 Feb 10;12(2):e1005788. doi: 10.1371/journal.pgen.1005788 (PMC4749232; doi:10.1371/journal.pgen.1005788)
Supplement: S9 Table — (PDF) [file pgen.1005788.s017.pdf]

Amrit et al., Table S9: Effect of mutations in TCER-1-Specific DOWN genes on lifespan

Strain	Gene	Trial 1				Trial 2			
		n = obs/ total	Mean +/- SEM	% Change *vs.* N2 lifespan	p *(vs N2)*	n = obs/ total	Mean +/- SEM	% Change *vs.* N2 lifespan	p *(vs N2)*
N2	***Control***	68/79	20.2 +/- 0.4			67/77	17.7 +/- 0.4		
RB2248	***gst-24(ok2042)***	52/75	23.6 +/- 0.7	16.6	0.0001	71/89	18.7 +/- 0.7	5.9	0.0022
RB2200	***gst-24(ok2980)***	78/94	21.4 +/- 0.4	5.6	0.1054	61/70	16.5 +/- 0.5	-6.7	0.1
RB1749	***numr-1(ok2239)***	51/79	22.4 +/- 0.5	10.7	0.0007	57/79	18.1 +/- 0.1	2.2	0.1
OH4240*	***lin-17(ot260)***	13/66	19.5 +/- 0.2	-3.7	0.7623	82/92	8.34 +/- 0.3	-52.8	<0.0001
RB2283	***lys-4(ok3106)***	66/93	21.8 +/- 0.5	7.9	0.0203	71/76	16.8 +/- 0.6	-4.6	0.78
OH4247**	***dopy-6(ot263)***	45/100	26.5 +/- 1.3	30.8	1.30E-06	76/107	20.4 +/- 0.7	15.4	0.0001

Complete genotypes: **lin-17(ot260) I; vtIs1 [dat-1p::GFP + rol-6]* *** vtIs1[dat-1p::GFP + rol-6] V; dopy-6(ot263) X.*
