# Supplementary material for: DAF-16 and TCER-1 Facilitate Adaptation to Germline Loss by Restoring Lipid Homeostasis and Repressing Reproductive Physiology in C. elegans
Source: PLoS Genet. 2016 Feb 10;12(2):e1005788. doi: 10.1371/journal.pgen.1005788 (PMC4749232; doi:10.1371/journal.pgen.1005788)
Supplement: S10 Table — (PDF) [file pgen.1005788.s018.pdf]

Amrit et al., Table S10: Effect of RNAi inactivation of lipid-metabolic genes on lifespan of wild-type worms

Wb Gene ID	Gene Name	Trial 1				Trial 2			
		n = obs/ total	Mean +/ SEM	% Change in N2 Lifespan	p (vs pAD12)	n = obs/ total	Mean +/ SEM	% Change in N2 Lifespan	p (vs pAD12)
**Control Vector**	**pAD12**	63/77	16.6 +/- 0.5			73/80	19.9 +/- 0.6		
**WBGene00000912**	***daf-16***	71/72	12.8 +/- 0.2	-22.8	<0.0001	92/100	15.1 +/- 0.3	-24.3	<0.0001
**WBGene00022855**	***tcer-1***	77/85	15.8 +/- 0.3	-4.6	0.0061	80/90	17.2 +/- 0.5	-13.5	0.0005
**WBGene00004076**	***pod-2***	60/105	10 +/- 0.1	-39.7	<0.0001	82/85	11.9 +/- 0.2	-40.2	<0.0001
**WBGene00009439**	***mlcd-1***	65/74	16.3 +/- 0.4	-1.6	0.4615	95/100	16.8 +/- 0.4	-15.8	0.0001
**WBGene00009342**	***fasn-1***	79/83	7.95 +/- .03	-52.1	<0.0001	59/90	15.8 +/- 0.3	-20.9	<0.0001
**WBGene00010296**	***dgat-2***	47/67	15.7 +/- 0.3	-5.2	0.0159	72/85	18.5 +/- 0.5	-7.2	<0.0001
**WBGene00021818**	**Y53G8B.2**	78/102	14.3 +/- 0.2	-13.3	<0.0001	61/88	17.6 +/- 0.4	-11.6	0.0008
**WBGene00017012**	***acs-22***	71/85	14.5 +/- 0.2	-12.2	<0.0001	81/85	15.9 +/- 0.5	-20	<0.0001
**WBGene00019464**	**K07B1.4**	61/84	15.1 +/- 0.3	-8.7	0.0077	65/98	18.3 +/- 0.4	-8.1	0.0123
**WBGene00010062**	***lipl-1***	69/76	14.4 +/- 0.2	-12.7	<0.0001	58/77	17.8 +/- 0.5	-10.8	0.0032
**WBGene00009773**	***lipl-2***	75/79	14.4 +/- 0.3	-12.8	<0.0001	62/77	18 +/- 0.4	-9.9	0.0046
**WBGene00022642**	***lipl-5***	76/99	14.8 +/- 0.3	-10.5	0.0001	64/86	17.8 +/- 0.4	-10.8	0.0013
**WBGene00008803**	***lips-10***	61/74	15.6 +/- 0.4	-5.9	0.0095	53/69	19.1 +/- 0.5	-4.1	0.1853
**WBGene00019208**	***lips-14***	59/83	15.1 +/- 0.3	-8.6	0.001	75/96	16.8 +/- 0.4	-15.7	<0.0001
**WBGene00015484**	***atgl-1***	62/76	15.6 +/- 0.4	-5.6	0.0329	89/95	16.3 +/- 0.5	-18.2	0.0001
