# Supplementary material for: DAF-16 and TCER-1 Facilitate Adaptation to Germline Loss by Restoring Lipid Homeostasis and Repressing Reproductive Physiology in C. elegans
Source: PLoS Genet. 2016 Feb 10;12(2):e1005788. doi: 10.1371/journal.pgen.1005788 (PMC4749232; doi:10.1371/journal.pgen.1005788)
Supplement: S11 Table — (PDF) [file pgen.1005788.s019.pdf]

Amrit et al., Table S11: Human homologs and orthologs of lipid-metabolic genes identified as DAF-16 and/or TCER-1 targets through RNA-Seq

#	Wormbase Gene ID	Worm Gene	Human Ortholog	Ensemble ID	Disease(s) linked to gene dysfunction
1	WBGene00004076	*pod-2*	ACACA: Acetyl-CoA carboxylase alpha<br>ACACB: Acetyl-CoA carboxylase beta	ENSG00000278540; ENSG00000076555	Fatty liver, insulin resistance [1,2]
2	WBGene00009439	*mlcd-1*	MLYCD: Malonyl-CoA decarboxylase	ENSG00000260300; ENSG00000103150	Malonyl-CoA decarboxylase deficiency, malonic aciduria, cardiomyopathy [3,4]
3	WBGene00009342	*fasn-1*	FASN: Fatty acid synthase	ENSG00000169710	Fatty liver, breast cancer, prostate cancer, insulin resistance [5]
4	WBGene00010408	*mboa-2*	DGAT1: Diacylglycerol O-acyltransferase 1	ENSG00000185000	Congenital diarrheal disorder, insulin resistance [6]
5	WBGene00010296	*dgat-2*	DGAT2: Diacylglycerol O-acyltransferase 2	ENSG00000062282	Alcoholic fatty liver disease [7]
6	WBGene00017012	*acs-22*	SLC27A4/ACSVL4/FATP4	ENSG00000130304; ENSG00000167114	Ichtyosis prematurity syndrome, insulin resistance [8]
7	WBGene00021818	Y53G8B.2	DGAT2, DGAT2L6, AWAT1, AWAT2, MOGAT1, MOGAT2 and MOGAT3	ENSG00000166391; ENSG00000184210; ENSG00000204195; ENSG00000062282; ENSG00000106384; ENSG00000147160; ENSG00000124003	Alcoholic fatty liver disease [7]
8	WBGene00019464	K07B1.4			
9	WBGene00001397	*fat-5*	SCD, SCD5	ENSG00000099194; ENSG00000145284	Spondylocostal dysostosis, faty liver disease, cvd, metabolic disorder, neoplastic transformation [9,10]
10	WBGene00001398	*fat-6*			
11	WBGene00001399	*fat-7*			
12	WBGene00010062	*lipl-1*	Gastric triacylglycerol lipase isoform 1, lipase family members A, M, J, K and N (LIPA, LIPM,LIPJ, LIPK and LIPN, respectively)	ENSG00000173239; ENSG00000107798; ENSG00000204020; ENSG00000204021; ENSG00000182333;	Wolman disease, cholesteryl ester storage disease, lysosomal storage diseases, autosomal recessive congenital ichthyosis, atherosclerosis [11,12,13,14]
13	WBGene00009773	*lipl-2*			
14	WBGene00022642	*lipl-5*			
15	WBGene00015484	*atgl-1*	PNPLA1: Patatin-like phospholipase domain containing 1<br>PNPLA2: Patatin-like phospholipase domain containing 2<br>PNPLA3: Patatin-like phospholipase domain containing 3	ENSG00000100341; ENSG00000100344; ENSG00000177666; ENSG00000180316	neutral lipid storage disease, Nonalcoholic fatty liver disease (NAFLD), familial hyperlipidemia [15,16]
16	WBGene00016716	*acs-17*	ACSL4: Acyl CoA synthetase	ENSG00000123983; ENSG00000068366	Alport Syndrome, hereditary elliptocytosis, mental retardation, adenocarcinoma [17,18]
17	WBGene00011122	*cpt-2*	CPT2: Carnitine palmitoyltransferase II	ENSG00000157184	Carnitine palmitoyltransferase II (CPT II) deficiency, infection-induced acute encephalopathy, metabolic disorder [19]
18	WBGene00017874	*acdh-9*	ACAD8: Acyl-CoA dehydrogenase family, member 8	ENSG00000151498	isobutyryl-CoA dehydrogenase (IBD) deficiency [20]
19	WBGene00001156	*ech-7*	ECHS1: Short-chain enoyl-CoA hydratase	ENSG00000127884	Short-chain enoyl-CoA hydratase 1 deficiency (fatal neurodegenerative disorder), Leigh syndrome [21,22]
20	WBGene00020347	*ech-1.2*	HADHA: Hydroxyacyl-CoA Dehydrogenase/3-Ketoacyl-CoA Thiolase/Enoyl-CoA Hydratase (Trifunctional Protein), Alpha Subunit	ENSG00000084754	Mitochondrial trifunctional protein (MTP) deficiency, LCHAD deficiency, HELLP syndrome, reye-like syndrome, fatty liver, cardiomyopathy [23,24]
21	WBGene00008564	*acox-1*	ACOX1: Peroxisomal straight-chain acyl-CoA oxidase 1	ENSG00000161533; ENSG00000168306	Peroxisomal acyl-CoA oxidase deficiency, Pseudoneonatal adrenoleukodystrophy [25,26]
22	WBGene00008565	F08A8.2	ACOX2: Peroxisomal acyl-coenzyme A oxidase 2		
23	WBGene00008566	F08A8.3			
24	WBGene00008567	F08A8.4			
25	WBGene00008167	C48B4.1			
26	WBGene00009952	*acaa-2*	ACAA2: Acetyl-CoA acetyltransferase	ENSG00000167315	Beta ketothiolase deficiency; Diabetes Mellitus, Type 2; osteoarthritis [27,28,29]
27	WBGene00019978	*hacd-1*	HADH: Hydroxyacyl-coenzyme A dehydrogenase		Hyperinsulinism, hypoglycemia [30]
28	WBGene00001394	*fat-2*			
29	WBGene00009221	*acs-2*	Similar to ACSF2: Acyl-CoA synthetase 2		
30	WBGene00001239	*elo-1*	Similar to ELOVL fatty acid elongase		
31	WBGene00001240	*elo-2*			
32	WBGene00012860	*acdh-11*	Highly similar to very-long-chain specific acyl-CoA dehydrogenase		
33	WBGene00008803	*lips-10*			
34	WBGene00019208	*lips-14*			
