# Supplementary material for: DAF-16 and TCER-1 Facilitate Adaptation to Germline Loss by Restoring Lipid Homeostasis and Repressing Reproductive Physiology in C. elegans
Source: PLoS Genet. 2016 Feb 10;12(2):e1005788. doi: 10.1371/journal.pgen.1005788 (PMC4749232; doi:10.1371/journal.pgen.1005788)
Supplement: S12 Table — (PDF) [file pgen.1005788.s020.pdf]

Amrit et al., Table S12: Primers used in this study for Q-PCR assays

Gene	Cosmid	Primer Sequence		*Gene*	Cosmid	Primer Sequence	
ech-7	Y105E8A.4	Forward Primer	5' GAGAATGACTATTTCACGAACTGG 3'	*elo-1*	F56H11.4	Forward Primer	5' GTTACTATTCAGGCTTCAATCCTG 3'
		Reverse Primer	5' CTAAGGCAAATCCGTTGACAG 3'			Reverse Primer	5' TTGGAATGGTTGACGATTACG 3'
elo-2	F11E6.5	Forward Primer	5' TCACTGTTCTCGGGAATCG 3'	*acs-22*	D1009.1	Forward Primer	5' TTGAATCAGGTGAAACAGCA 3'
		Reverse Primer	5' AGTAGGAAGCGACAAACCC 3'			Reverse Primer	5' CAATTCTTGATACGTCAACTGC 3'
fat-5	W06D12.3	Forward Primer	5' TCTACACATTGGAGCCCTG 3'	*atgl-1*	C05D11.7	Forward Primer	5' GATCGACCGATGATTTATCGAG 3'
		Reverse Primer	5' CAGGAGGAATACCCATCCA 3'			Reverse Primer	5' GAGCCAATCCACATTTGGT 3'
pod-2	W09B6.1	Forward Primer	5' GAGCAGTATTACGAGACGC 3'	*fasn-1*	F32H2.5	Forward Primer	5' GGATAATACTGGAGAAGGATCG 3'
		Reverse Primer	5' GAGCTGCTGTTCATATCGT 3'			Reverse Primer	5' ATGTTGTCCGAAGACTGAG 3'
acs-2	F28F8.2	Forward Primer	5' TCCGGATAAGGAGTTCTGTG 3'	*lipl-1*	F54F3.3	Forward Primer	5' GGACTTAAAGTTGAAGCTGGAG 3'
		Reverse Primer	5' ATTTGACGGACGTCATGGT 3'			Reverse Primer	5' AACACGAGTTGCGTTAAGC 3'
mlcd-1	F35G12.1	Forward Primer	5' ATTCAGTTGCAAACTTCCAC 3'	*lipl-2*	F46B6.8	Forward Primer	5' GTTACATGGCCAAATGGGA 3'
		Reverse Primer	5' TCCCTCTATGACTCGTATCTC 3'			Reverse Primer	5' AAACGAAAGCTGCACTCTG 3'
	F53A2.7	Forward Primer	5' ATCTTCGAGGTAAATGAGGC 3'	*lipl-5*	ZK6.7	Forward Primer	5' CAAGAAGTTCTTCGCGCTG 3'
		Reverse Primer	5' CCACCATTGACATTCAGCT 3'			Reverse Primer	5' CGAACCATCCATCAAACTCC 3'
dgat-2	F59A1.10	Forward Primer	5' TCGGATATCATCCTCATGGA 3'	*lips-10*	F14E5.5	Forward Primer	5' CGATTATGTGTTGAAGGTTCG 3'
		Reverse Primer	5' ATGCCTTTGAACACTTGCT 3'			Reverse Primer	5' GAGCAAGCAATGATGTTTACTG 3'
cpt-2	R07H5.2	Forward Primer	5' GCAAGATGGATTCGGTATTGG 3'	*lips-14*	H17B01.3	Forward Primer	5' TTGGGCTAGGAATAGCCAG 3'
		Reverse Primer	5' TCACGTTTCGACTTATTGCTC 3'			Reverse Primer	5' TGAGTAGACGATAGACTTGGTG 3'
acdh-11	Y45F3A.3	Forward Primer	5' AATACAGCCACGCAAAGAC 3'	*mboa-2*	H19N07.4	Forward Primer	5' GAAATGAGAGGACCTTGCG 3'
		Reverse Primer	5' CGAGAATTGGGTCATCTTTATAAGG 3'			Reverse Primer	5' GAAATTTGTCCATCCAGAACTC 3'
acs-17	C46F4.2	Forward Primer	5' TATGCTCAGGTTGACAAACG 3'	*nhr-49*	K10C3.6	Forward Primer	5' TTGGCAGAGGTGGATTCTC 3'
		Reverse Primer	5' AATAATTGGCTCCTTCTCCTG 3'			Reverse Primer	5' CTGTAAAGAGACCGGAGCC 3'
hacd-1	R09B5.6	Forward Primer	5' ATACTGATATTCCGTCCGCTG 3'		Y45F10D.4	Forward Primer	5' TTCACTGTTCAATGCTCGC 3'
		Reverse Primer	5' GGAAGATGTCAAGTTTCAGATCC 3'			Reverse Primer	5' CTTAGGCCTTCTTAGTCTGCT 3'
	Y53G8B.2	Forward Primer	5' TAGCACTTCAGTTCAGAATGG 3'	*hlh-30*	W02C12.3	Forward Primer	5' ACAATATGATTGAACGCCGAC 3'
		Reverse Primer	5' CAATGGATTCTCTGGAGCA 3'			Reverse Primer	5' TCTGATGTGTTCTTCGGCA 3'
ech-1.2	T08B2.7	Forward Primer	5' TGGATAAGCTCCAATCCGA 3'	*daf-12*	F11A1.3	Forward Primer	5' CCAAACATTATGGCTCATCGT 3'
		Reverse Primer	5' ATTTGGATGTCAGCTCCTG 3'			Reverse Primer	5' GGAAAGAACAACCTGTCCAC 3'
skn-1	T19E7.2	Forward Primer	5' ACCAAACGATGTGTTCCCA 3'	*mdt-15*	R12B2.5	Forward Primer	5' AATGGACCACCTGGTAGTG 3'
		Reverse Primer	5' CTGTCCATTTGATACAACTGCT 3'			Reverse Primer	5' ACGAGCTTGATCCATATCCT 3'
atfs-1	ZC376.7	Forward Primer	5' CCGATCGAAGACGAATGTTG 3'	*rpl-32*	T24B8.1	Forward Primer	5' GGATTTGGACATGCTCCTC 3'
		Reverse Primer	5' GAACTCCATTTTTCTTCCC 3'			Reverse Primer	5' GATTCCCTTGCGGCTCTT 3'
