# Supplementary material for: DAF-16 and TCER-1 Facilitate Adaptation to Germline Loss by Restoring Lipid Homeostasis and Repressing Reproductive Physiology in C. elegans
Source: PLoS Genet. 2016 Feb 10;12(2):e1005788. doi: 10.1371/journal.pgen.1005788 (PMC4749232; doi:10.1371/journal.pgen.1005788)
Supplement: S1 Text — (PDF) [file pgen.1005788.s021.pdf]

Supplementary References: References associated with S11 Table

1. Phillips CM, Goumidi L, Bertrais S, Field MR, Cupples LA, Ordovas JM, et al. ACC2 gene polymorphisms, metabolic syndrome, and gene-nutrient interactions with dietary fat. J Lipid Res. 2010;51(12):3500-7. doi: 10.1194/jlr.M008474. PubMed PMID: 20855566; PubMed Central PMCID: PMC2975722.

2. Riancho JA, Vazquez L, Garcia-Perez MA, Sainz J, Olmos JM, Hernandez JL, et al. Association of ACACB polymorphisms with obesity and diabetes. Mol Genet Metab. 2011;104(4):670-6. doi: 10.1016/j.ymgme.2011.08.013. PubMed PMID: 21908218.

3. FitzPatrick DR, Hill A, Tolmie JL, Thorburn DR, Christodoulou J. The molecular basis of malonyl-CoA decarboxylase deficiency. Am J Hum Genet. 1999;65(2):318-26. doi: 10.1086/302492. PubMed PMID: 10417274; PubMed Central PMCID: PMC1377930.

4. Ficicioglu C, Chrisant MR, Payan I, Chace DH. Cardiomyopathy and hypotonia in a 5-month-old infant with malonyl-coa decarboxylase deficiency: potential for preclinical diagnosis with expanded newborn screening. Pediatr Cardiol. 2005;26(6):881-3. doi: 10.1007/s00246-005-1045-x. PubMed PMID: 16078122.

5. Menendez JA, Vazquez-Martin A, Ortega FJ, Fernandez-Real JM. Fatty acid synthase: association with insulin resistance, type 2 diabetes, and cancer. Clin Chem. 2009;55(3):425-38. doi: 10.1373/clinchem.2008.115352. PubMed PMID: 19181734.

6. Haas JT, Winter HS, Lim E, Kirby A, Blumenstiel B, DeFelice M, et al. DGAT1 mutation is linked to a congenital diarrheal disorder. J Clin Invest. 2012;122(12):4680-4. doi: 10.1172/JCI64873. PubMed PMID: 23114594; PubMed Central PMCID: PMC3533555.

7. Wang Z, Yao T, Song Z. Involvement and mechanism of DGAT2 upregulation in the pathogenesis of alcoholic fatty liver disease. J Lipid Res. 2010;51(11):3158-65. doi: 10.1194/jlr.M007948. PubMed PMID: 20739640; PubMed Central PMCID: PMC2952556.

8. Klar J, Schweiger M, Zimmerman R, Zechner R, Li H, Torma H, et al. Mutations in the fatty acid transport protein 4 gene cause the ichthyosis prematurity syndrome. Am J Hum Genet. 2009;85(2):248-53. doi: 10.1016/j.ajhg.2009.06.021. PubMed PMID: 19631310; PubMed Central PMCID: PMC2725242.

9. Kotronen A, Seppanen-Laakso T, Westerbacka J, Kiviluoto T, Arola J, Ruskeepaa AL, et al. Hepatic stearoyl-CoA desaturase (SCD)-1 activity and diacylglycerol but not ceramide concentrations are increased in the nonalcoholic human fatty liver. Diabetes. 2009;58(1):203-8. doi: 10.2337/db08-1074. PubMed PMID: 18952834; PubMed Central PMCID: PMC2606873.

10. Attie AD, Krauss RM, Gray-Keller MP, Brownlie A, Miyazaki M, Kastelein JJ, et al. Relationship between stearoyl-CoA desaturase activity and plasma triglycerides in human and mouse hypertriglyceridemia. J Lipid Res. 2002;43(11):1899-907. PubMed PMID: 12401889.

11. Anderson RA, Byrum RS, Coates PM, Sando GN. Mutations at the lysosomal acid cholesteryl ester hydrolase gene locus in Wolman disease. Proceedings of the National Academy of Sciences of the United States of America. 1994;91(7):2718-22. PubMed PMID: 8146180; PubMed Central PMCID: PMC43441.

12. Israeli S, Khamaysi Z, Fuchs-Telem D, Nousbeck J, Bergman R, Sarig O, et al. A mutation in LIPN, encoding epidermal lipase N, causes a late-onset form of autosomal-recessive congenital ichthyosis. Am J Hum Genet. 2011;88(4):482-7. doi: 10.1016/j.ajhg.2011.02.011. PubMed PMID: 21439540; PubMed Central PMCID: PMC3071911.

13. Pagani F, Garcia R, Pariyarath R, Stuani C, Gridelli B, Paone G, et al. Expression of lysosomal acid lipase mutants detected in three patients with cholesteryl ester storage disease. Hum Mol Genet. 1996;5(10):1611-7. PubMed PMID: 8894696.

14. Zschenker O, Illies T, Ameis D. Overexpression of lysosomal acid lipase and other proteins in atherosclerosis. J Biochem. 2006;140(1):23-38. doi: 10.1093/jb/mvj137. PubMed PMID: 16877765.

15. Fischer J, Lefevre C, Morava E, Mussini JM, Laforet P, Negre-Salvayre A, et al. The gene encoding adipose triglyceride lipase (PNPLA2) is mutated in neutral lipid storage disease with myopathy. Nature genetics. 2007;39(1):28-30. doi: 10.1038/ng1951. PubMed PMID: 17187067.

16. Nanni L, Quagliarini F, Megiorni F, Montali A, Minicocci I, Campagna F, et al. Genetic variants in adipose triglyceride lipase influence lipid levels in familial combined hyperlipidemia. Atherosclerosis. 2010;213(1):206-11. doi: 10.1016/j.atherosclerosis.2010.08.055. PubMed PMID: 20832801.

17. Piccini M, Vitelli F, Bruttini M, Pober BR, Jonsson JJ, Villanova M, et al. FACL4, a new gene encoding long-chain acyl-CoA synthetase 4, is deleted in a family with Alport syndrome, elliptocytosis, and mental retardation. Genomics. 1998;47(3):350-8. doi: 10.1006/geno.1997.5104. PubMed PMID: 9480748.

18. Cao Y, Dave KB, Doan TP, Prescott SM. Fatty acid CoA ligase 4 is up-regulated in colon adenocarcinoma. Cancer Res. 2001;61(23):8429-34. PubMed PMID: 11731423.

19. Thuillier L, Rostane H, Droin V, Demaugre F, Brivet M, Kadhom N, et al. Correlation between genotype, metabolic data, and clinical presentation in carnitine palmitoyltransferase 2 (CPT2) deficiency. Hum Mutat. 2003;21(5):493-501. doi: 10.1002/humu.10201. PubMed PMID: 12673791.

20. Nguyen TV, Andresen BS, Corydon TJ, Ghisla S, Abd-El Razik N, Mohsen AW, et al. Identification of isobutyryl-CoA dehydrogenase and its deficiency in humans. Mol Genet Metab. 2002;77(1-2):68-79. PubMed PMID: 12359132.

21. Sakai C, Yamaguchi S, Sasaki M, Miyamoto Y, Matsushima Y, Goto Y. ECHS1 mutations cause combined respiratory chain deficiency resulting in Leigh syndrome. Hum Mutat. 2015;36(2):232-9. doi: 10.1002/humu.22730. PubMed PMID: 25393721.

22. Peters H, Buck N, Wanders R, Ruiter J, Waterham H, Koster J, et al. ECHS1 mutations in Leigh disease: a new inborn error of metabolism affecting valine metabolism. Brain. 2014;137(Pt 11):2903-8. doi: 10.1093/brain/awu216. PubMed PMID: 25125611.

23. Dionisi Vici C, Burlina AB, Bertini E, Bachmann C, Mazziotta MR, Zacchello F, et al. Progressive neuropathy and recurrent myoglobinuria in a child with long-chain 3-hydroxyacyl-coenzyme A dehydrogenase deficiency. J Pediatr. 1991;118(5):744-6. PubMed PMID: 2019931.

24. Choi JH, Yoon HR, Kim GH, Park SJ, Shin YL, Yoo HW. Identification of novel mutations of the HADHA and HADHB genes in patients with mitochondrial trifunctional protein deficiency. Int J Mol Med. 2007;19(1):81-7. PubMed PMID: 17143551.

25. Ferdinandusse S, Denis S, Hogenhout EM, Koster J, van Roermund CW, L IJ, et al. Clinical, biochemical, and mutational spectrum of peroxisomal acyl-coenzyme A oxidase deficiency. Hum Mutat. 2007;28(9):904-12. doi: 10.1002/humu.20535. PubMed PMID: 17458872.

26. Johansson A, Curran JE, Johnson MP, Freed KA, Fenstad MH, Bjorge L, et al. Identification of ACOX2 as a shared genetic risk factor for preeclampsia and cardiovascular disease. Eur J Hum Genet. 2011;19(7):796-800. doi: 10.1038/ejhg.2011.19. PubMed PMID: 21343950; PubMed Central PMCID: PMC3137494.

27. Ruiz-Romero C, Calamia V, Mateos J, Carreira V, Martinez-Gomariz M, Fernandez M, et al. Mitochondrial dysregulation of osteoarthritic human articular chondrocytes analyzed by proteomics: a decrease in mitochondrial superoxide dismutase points to a redox imbalance. Mol Cell Proteomics. 2009;8(1):172-89. doi: 10.1074/mcp.M800292-MCP200. PubMed PMID: 18784066; PubMed Central PMCID: PMC2713027.

28. Fontaine-Bisson B, Renstrom F, Rolandsson O, Magic, Payne F, Hallmans G, et al. Evaluating the discriminative power of multi-trait genetic risk scores for type 2 diabetes in a northern Swedish population. Diabetologia. 2010;53(10):2155-62. doi: 10.1007/s00125-010-1792-y. PubMed PMID: 20571754; PubMed Central PMCID: PMC2931645.

29. Fukao T, Scriver CR, Kondo N, t2 Collaborative Working G. The clinical phenotype and outcome of mitochondrial acetoacetyl-CoA thiolase deficiency (beta-ketothiolase or T2 deficiency) in 26 enzymatically proved and mutation-defined patients. Mol Genet Metab. 2001;72(2):109-14. doi: 10.1006/mgme.2000.3113. PubMed PMID: 11161836.

30. Flanagan SE, Patch AM, Locke JM, Akcay T, Simsek E, Alaei M, et al. Genome-wide homozygosity analysis reveals HADH mutations as a common cause of diazoxide-responsive hyperinsulinemic-hypoglycemia in consanguineous pedigrees. The Journal of clinical endocrinology and metabolism. 2011;96(3):E498-502. doi: 10.1210/jc.2010-1906. PubMed PMID: 21252247; PubMed Central PMCID: PMC3100671.
